# Author Correction: TC10 regulates breast cancer invasion and metastasis by controlling membrane type-1 matrix metalloproteinase at invadopodia

**DOI:** 10.1038/s42003-021-02785-9

**Published:** 2021-10-26

**Authors:** Maren Hülsemann, Colline Sanchez, Polina V. Verkhusha, Vera Des Marais, Serena P. H. Mao, Sara K. Donnelly, Jeffrey E. Segall, Louis Hodgson

**Affiliations:** 1 Department of Anatomy and Structural Biology, Albert Einstein College of Medicine, Bronx, NY 10461 USA; 2 Gruss-Lipper Biophotonics Center, Albert Einstein College of Medicine, Bronx, NY 10461 USA; 3 Analytical Imaging Facility, Albert Einstein College of Medicine, Bronx, NY 10461 USA

**Keywords:** RHO signalling, Breast cancer, Cell invasion

Correction to: *Communications Biology* 10.1038/s42003-021-02583-3, published online 16 September 2021

In the original version of the Article, the authors mistakenly included a set of images from an unrelated experiment in Figure 7i.

The correct version of panel 7i is:
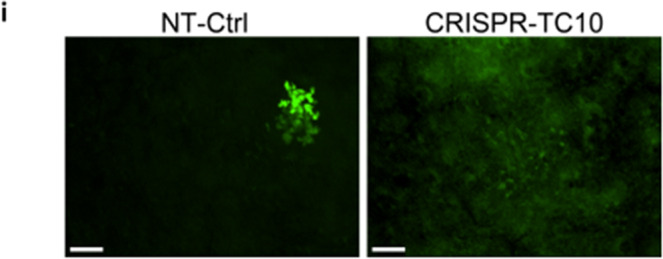


which replaces the previous incorrect version:
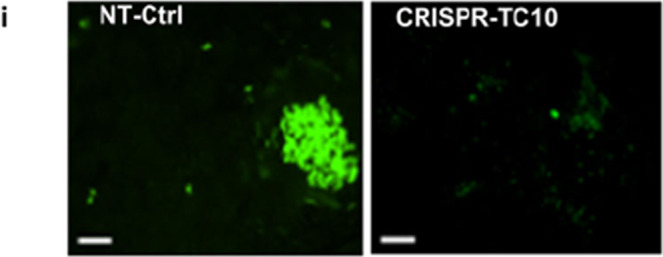


The figure has been corrected in both the PDF and HTML versions of the Article.

