# Supplementary material for: TC10 regulates breast cancer invasion and metastasis by controlling membrane type-1 matrix metalloproteinase at invadopodia
Source: Commun Biol. 2021 Sep 16;4:1091. doi: 10.1038/s42003-021-02583-3 (PMC8445963; doi:10.1038/s42003-021-02583-3)
Supplement: Supplementary file 1 — Supplementary Information [file 42003_2021_2583_MOESM1_ESM.pdf]

TC10 regulates breast cancer invasion and metastasis by controlling membrane type 1 matrix metalloproteinase at invadopodia

Maren Hülsemann [1,2], Colline Sanchez [1,2], Polina V. Verkhusha [1], Vera DesMarais [1,2,3], Serena P.H. Mao [1], Sara K. Donnelly [1], Jeffrey E. Segall [1,2], and Louis Hodgson [1,2,*]

Supplementary Figures

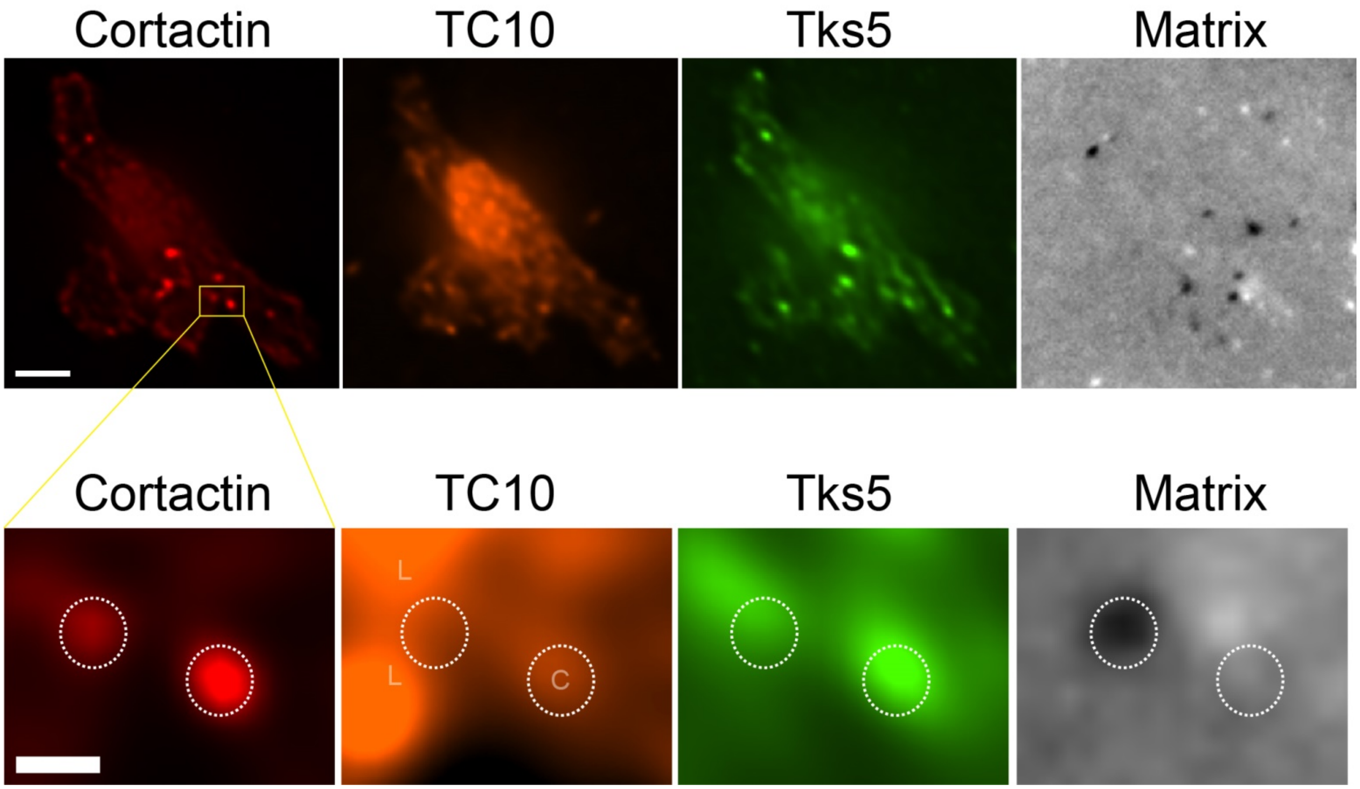

Supplementary Figure 1: A representative MDA-MB231 cells showing localization of endogenous TC10 at invadopodia. Upper panels show a single cell showing designated immunofluorescence staining. White bar = 5μm. Lower panels show a set of zoomed view of the yellow boxed region. Invadopodia are shown with white dotted circles. "L" indicates lateral localization of TC10, "C" indicates core localization of TC10. White bar = 1 μm.

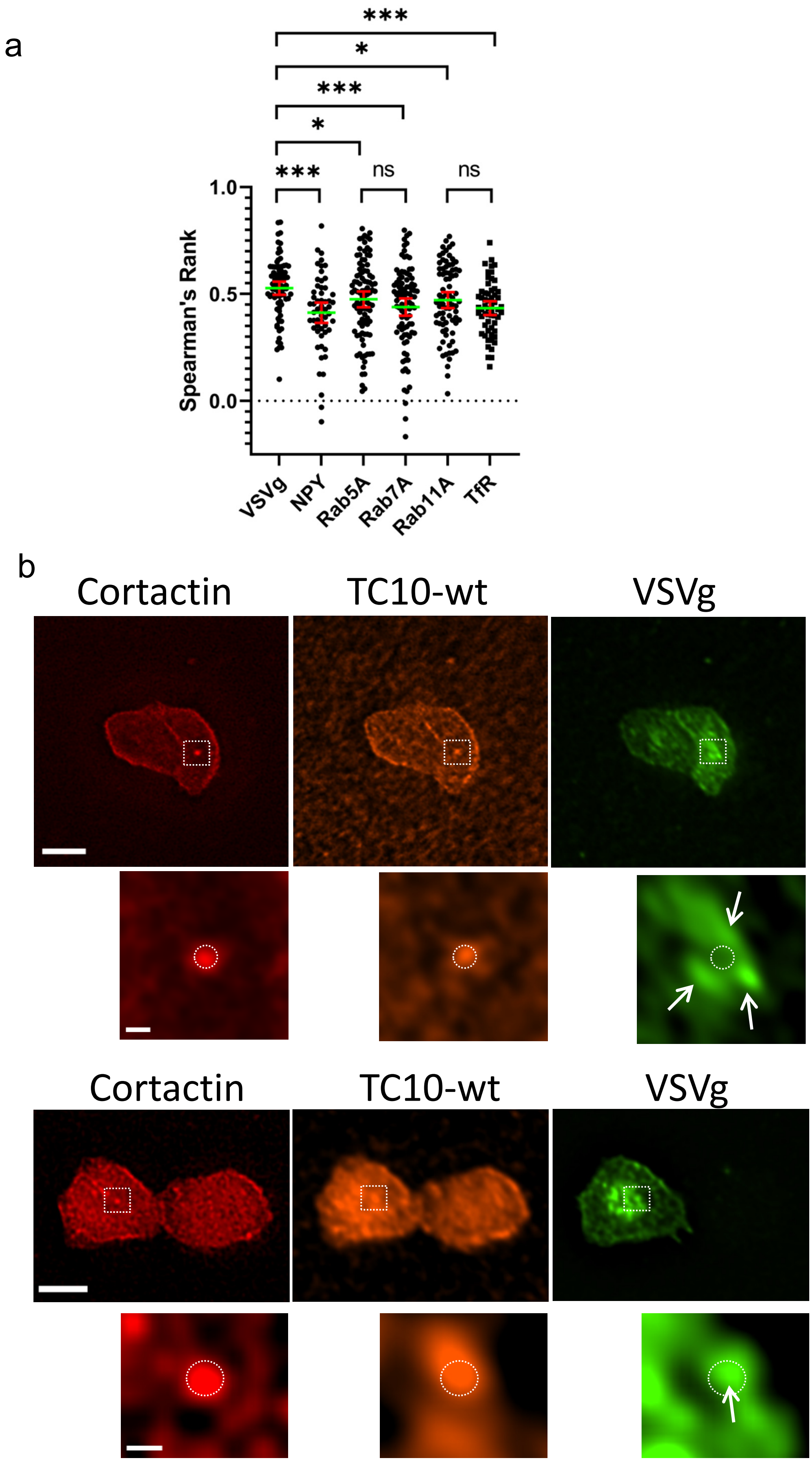
a
***
*
***
*
***
ns
ns
1.0
0.5
0.0
Spearman's Rank
VSVg
NPY
Rab5A
Rab7A
Rab11A
TfR
b
Cortactin
TC10-wt
VSVg
Cortactin
TC10-wt
VSVg

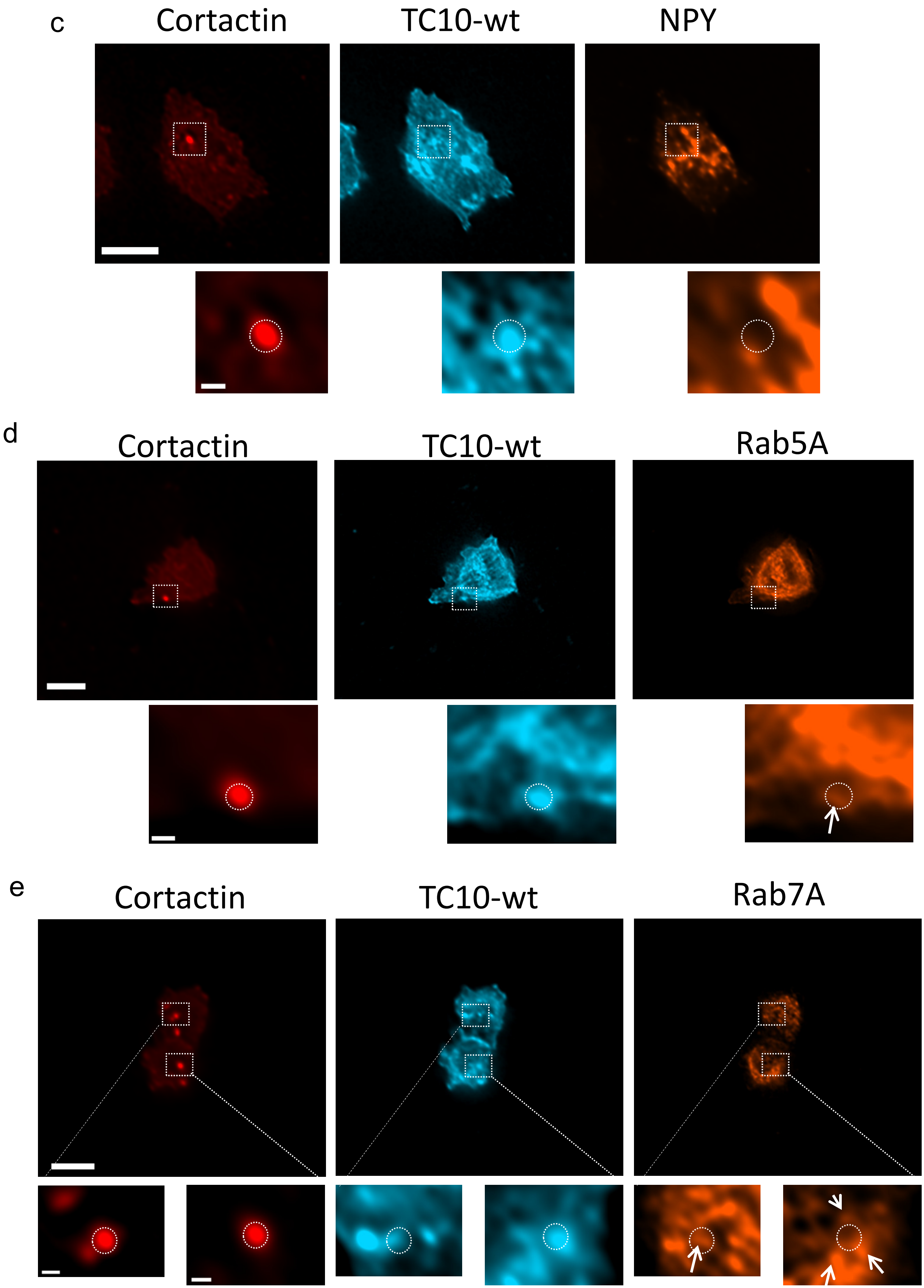

c
Cortactin
TC10-wt
NPY
d
Cortactin
TC10-wt
Rab5A
e
Cortactin
TC10-wt
Rab7A

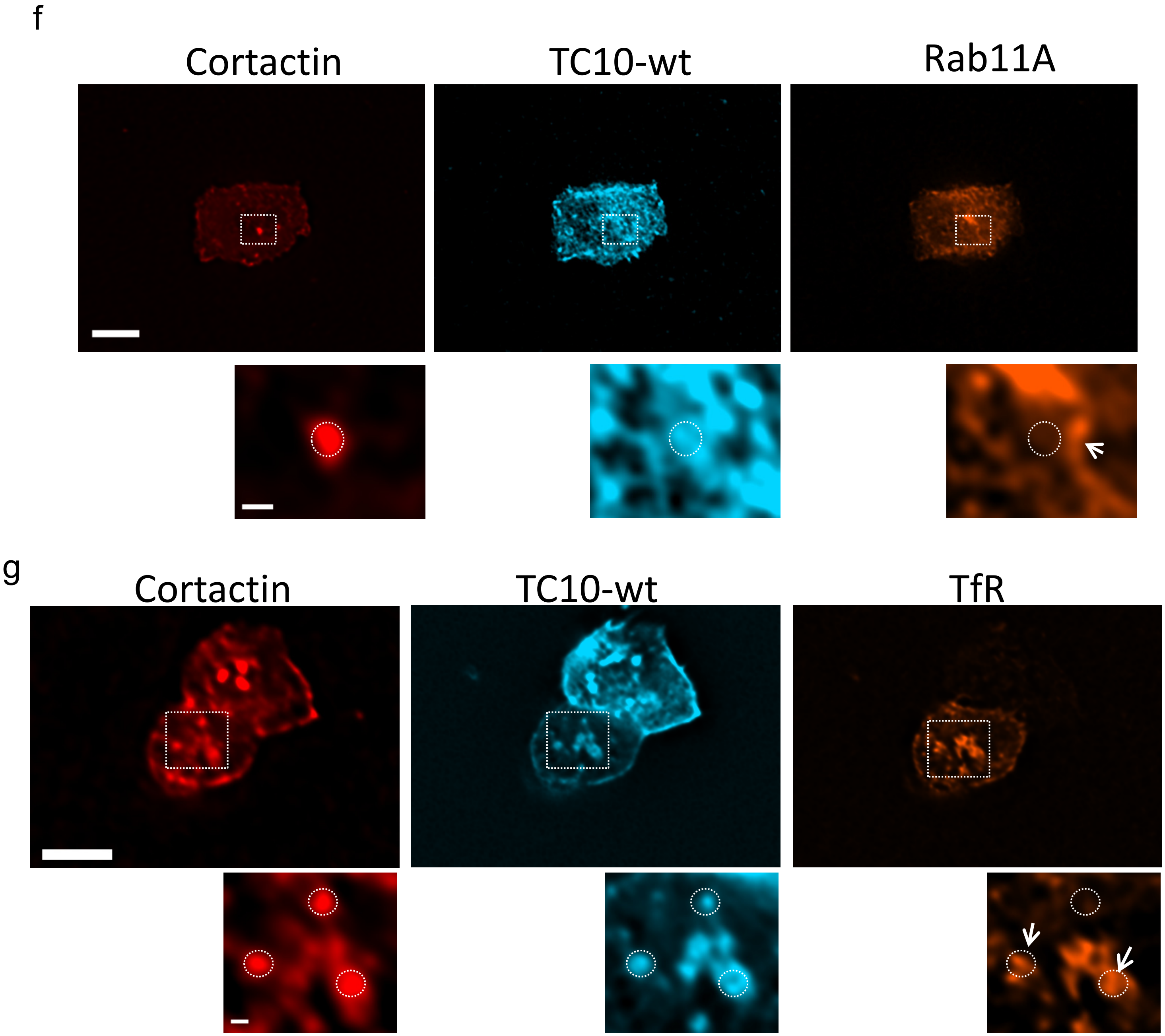

Supplementary Figure 2: Vesicular localization of TC10 in MTLn3 cells. **a.** Colocalization analysis between fluorescent protein tagged wildtype TC10 and the indicated vesicular compartment markers. Spearman's ranks were calcualted using Fiji-ImageJ using the Colc2 plugin module. VSVg vs. NPY: P=4.184x10^{-5}; VSVg vs. Rab5A: P=0.03651; VSVg vs. Rab7A: P=0.0009012; VSVg vs. Rab11A: P=0.02272; VSVg vs. TfR: P=6.930x10^{-5}; Rab5A vs. Rab7A: P=0.1985; Rab11A vs. TfR: P=0.1501. Student's t-test was used with unpaired, two-tailed analysis, n = 3 experiments, error bars represent 95% confidence interval of the data distribution around the mean. **b – g.** Representative images showing cortactin, TC10-WT and the vesicular markers, VSVg (**b**), NPY (**c**), Rab5A (**d**), Rab7A (**e**), Rab11A (**f**), and TfR (**g**), respectively. Core and/or side localizations of the vesicular markers in relation to the cortactin invadopodium core are shown with white arrows. White box in whole cell images represents the location of the zoomed view shown below each whole-cell views. White bars in the whole cell views are 10μm, and in the zoomed in views are 1μm. Dotted white circles in zoomed views show the locations of the invadopodia core marker cortactin spots.

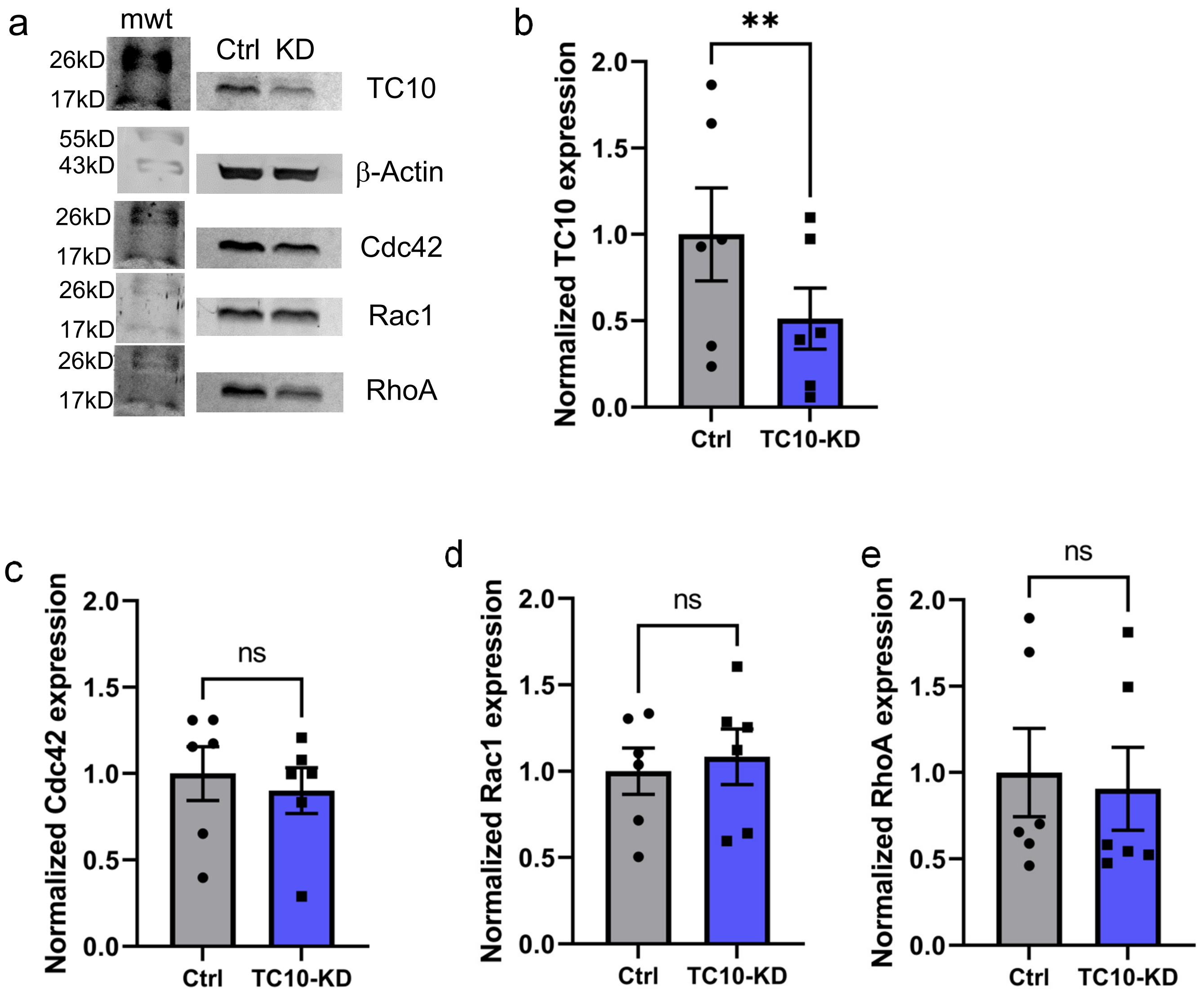

Supplementary Figure 3: TC10 depletion by siRNA in MTLn3 cells. **a.** Representative western blots showing Rho GTPase expression levels and the loading control. **b.** Quantification of TC10 levels in cells treated with TC10 siRNA; ** p = 0.003209. **c.** Quantification of Cdc42 levels in TC10-depleted cells; p = 0.2123. **d.** Quantification of Rac1 levels in TC10-depleted cells; p = 0.2420. **e.** Quantification of RhoA levels in TC10-depleted cells; p=0.1303. Student's t-test was used with paired, one-tailed analysis, n = 6 experiments, error bars represent SEM. Full size western blots are shown in Supplementary Figure 19. n.s., not significant; KD, knockdown. All quantifications are shown normalized against Ctrl.

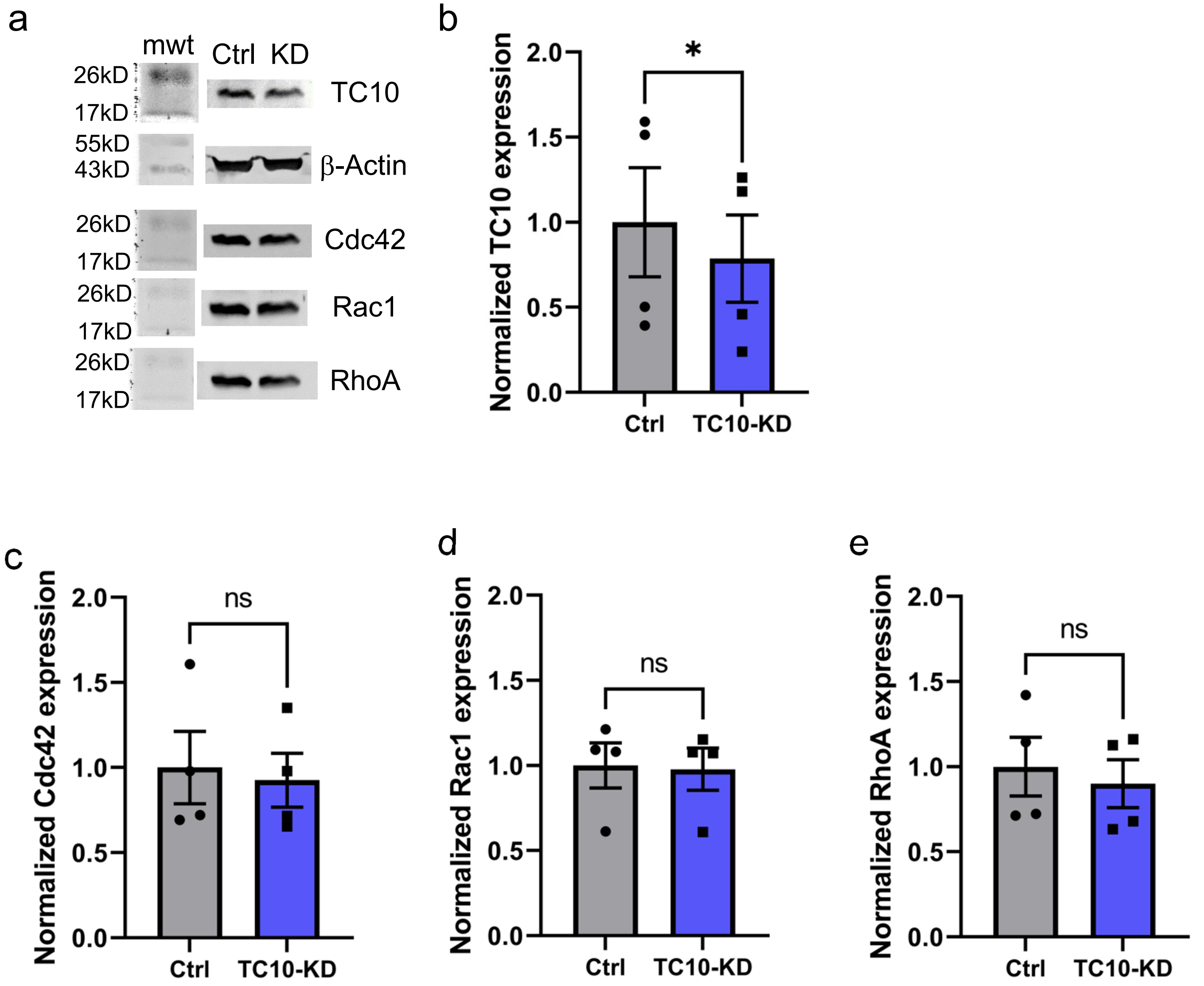

Supplementary Figure 4: TC10 depletion by siRNA in MDA-MB231 cells. **a.** Representative western blots showing the expression levels of Rho GTPases and the loading control. **b.** Quantification of TC10 levels in cells treated with TC10 siRNA; $^{*}p = 0.02838$. **c.** Quantification of Cdc42 levels in TC10-depleted cells; $p = 0.1546$. **d.** Quantification of Rac1 levels in TC10-depleted cells; $p = 0.1000$. **e.** Quantification of RhoA levels in TC10-depleted cells; $p = 0.1185$. Student's t-test was used with paired, one-tailed analysis, $n = 4$ experiments, error bars represent the SEM. Full size western blots are shown in Supplementary Figure 19. n.s., not significant; KD, knockdown. All quantifications are shown normalized against Ctrl.

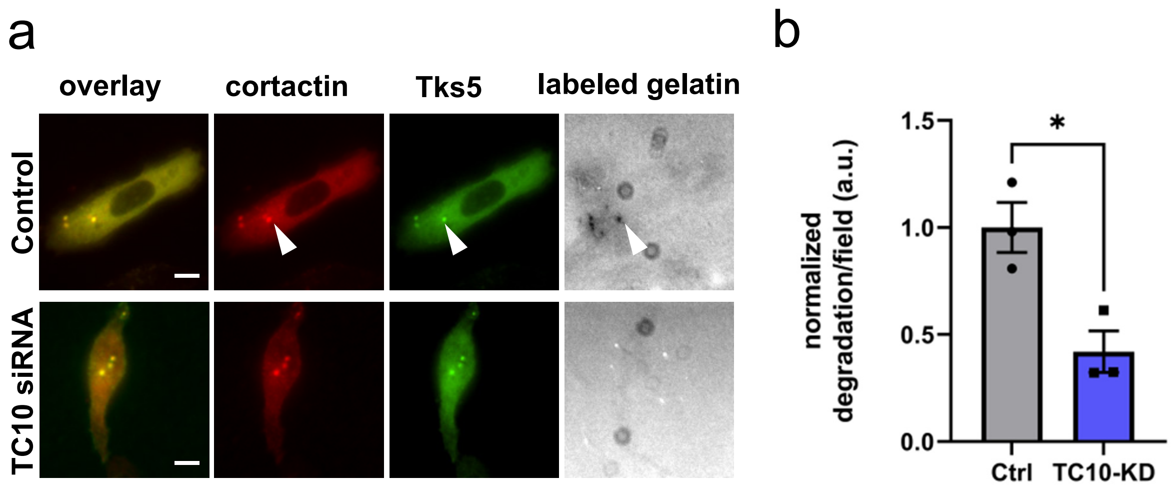

Supplementary Figure 5: TC10 depletion by siRNA in MDA-MB231 cells. **a**. Representative images from siRNA-mediated TC10 depletion in MDA–MB-231 cells impacting gelatin matrix degradation, visualized using a 405-nm fluorescent gelatin matrix. Invadopodia are denoted by cortactin and Tks5 colocalization with spots of matrix degradation (arrow). White bar = 10-µm. **b**. Quantification of the MDA-MB-231 matrix degradation when TC10 is depleted, shown in (**a**), normalized to the Ctrl. Student's t-test, one-tail analysis: * p=0.01528; n=3 experiments; shown with SEM.

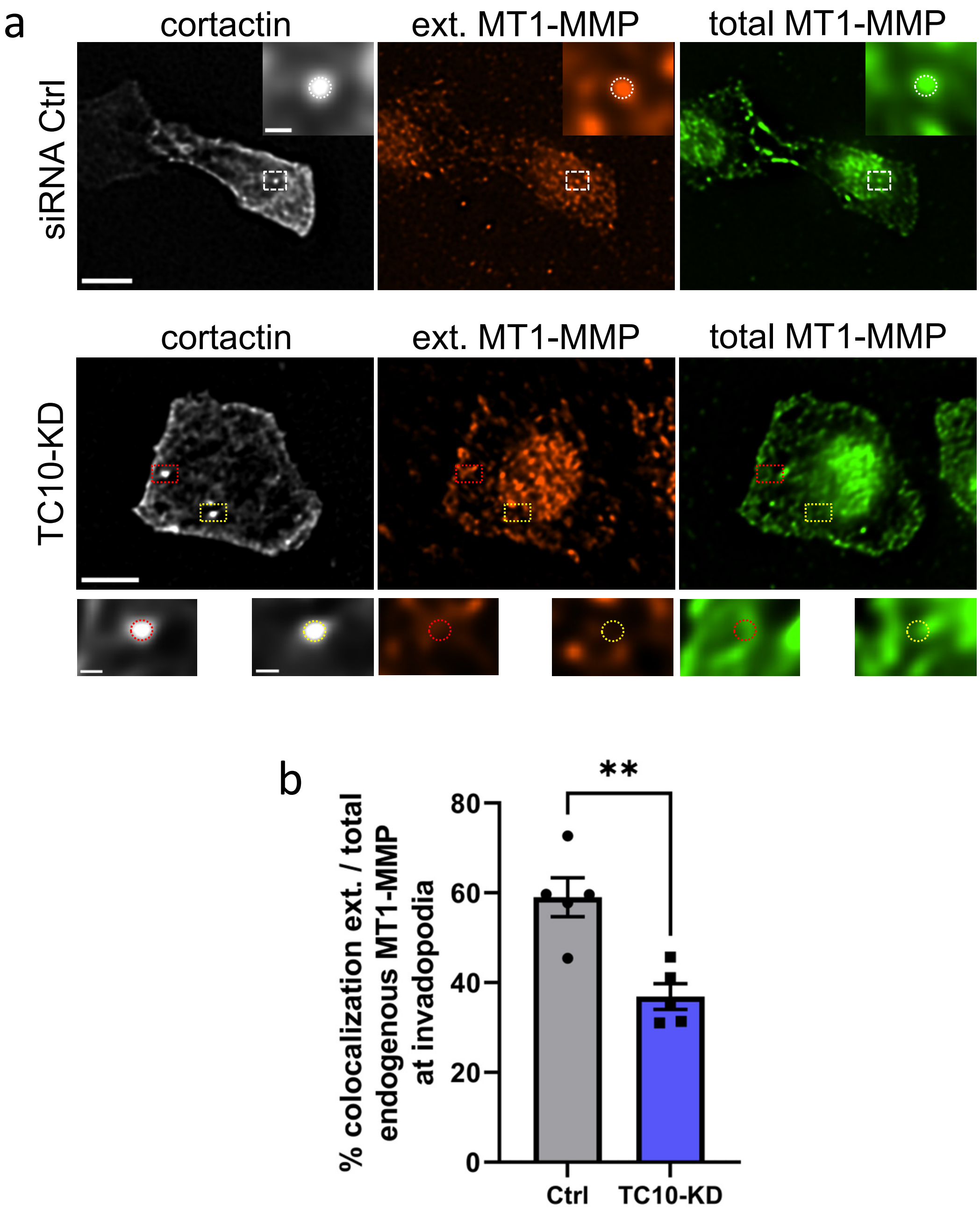

Supplementary Figure 6: TC10-depletion by siRNA in MTLn3 cells reduces the colocalization of externally stained endogenous MT1-MMP to the total endogenous MT1-MMP at invadopodia. **a**. Representative images from siRNA-mediated TC10 depletion in MTLn3 cells, stained for endogenous MT1-MMP using two different antibodies, with and without cell permeabilization. White bar in whole cell views = 10-μm, in zoomed views = 1-μm. The color-coded dashed boxes and circles indicate the respective locations of the zoomed views and the cortactin core locations. **b**. Quantification of (**a**). Student's t-test, paired two-tail analysis: ** p=0.001527; n=5 experiments; shown with SEM.

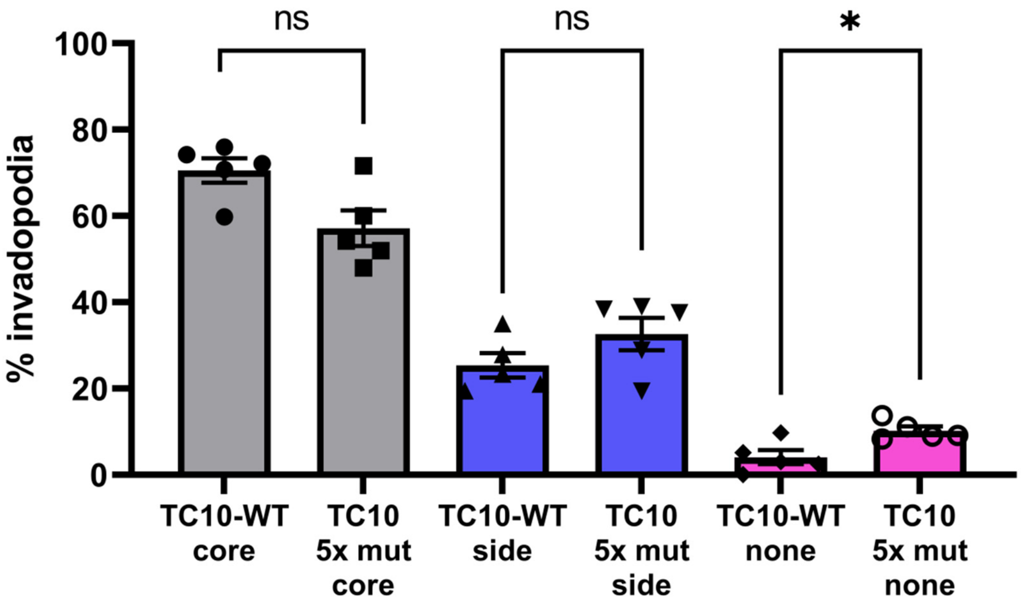

Supplementary Figure 7: Mutations in the Switch I/II effector binding regions of TC10 do not impact the spatial localization of TC10 in the invadopodia core versus the invadopodia side in MTLn3 cells, but a modest increase was observed in the non-targeted population of invadopodia. TC10 WT vs 5x mut: core, ns p = 0.0728; side, ns p = 0.293; none targeting, * p = 0.0372. N = 5 experiments consisting of at least 20 fields of view from each experiment, with ≥ 50 invadopodia from ≥ 25 cells for each condition. Comparisons were performed using Student's t-test with two-tailed paired analysis.

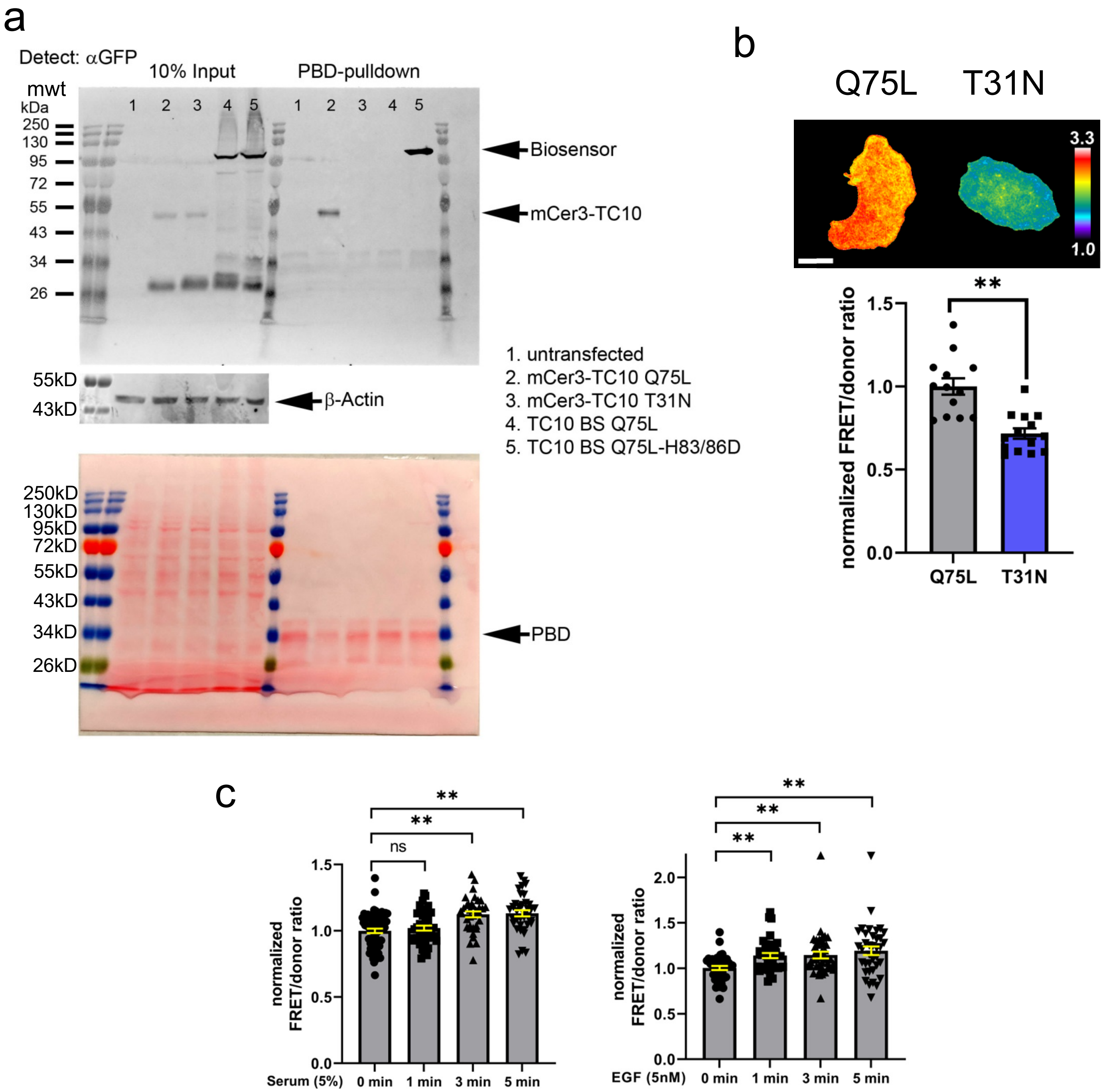

Supplementary Figure 8: Additional TC10 FRET biosensor characterizations. **a.** Competitive pull-down experiments. Lane designations are also shown. The constitutively activated TC10 biosensor was pulled down by excess exogenous GST-PBD, only when the GTPase binding mutations were present in both PBD1 and PBD2. A full blot for the loading control is shown in Supplementary Figure 19. **b.** A representative image set of the whole-cell average TC10 activity differences between the Q75L and T31N biosensor mutants expressed in MTLn3 cells, shown with quantification, normalized to the activated Q75L FRET/donor ratio. White bar = 20 μm. $^{**}p = 0.0000890$. Two-tailed Student's t-test, n = 13-14 cells imaged for each condition. **c.** TC10 biosensor response to serum or EGF stimulations following 4 h of serum starvation. For serum stimulation: 3 min, $^{**}p = 0.0000374$; 5 min, $^{**}p = 0.0000157$. For EGF stimulation: 1 min, $^{**}p = 0.000344$; 3 min, $^{**}p = 0.00181$; and 5 min, $^{**}p = 0.000587$. N = 5 experiments, analyzed by two-tailed Student's t-test. Results are normalized to t = 0-min FRET/donor ratio.

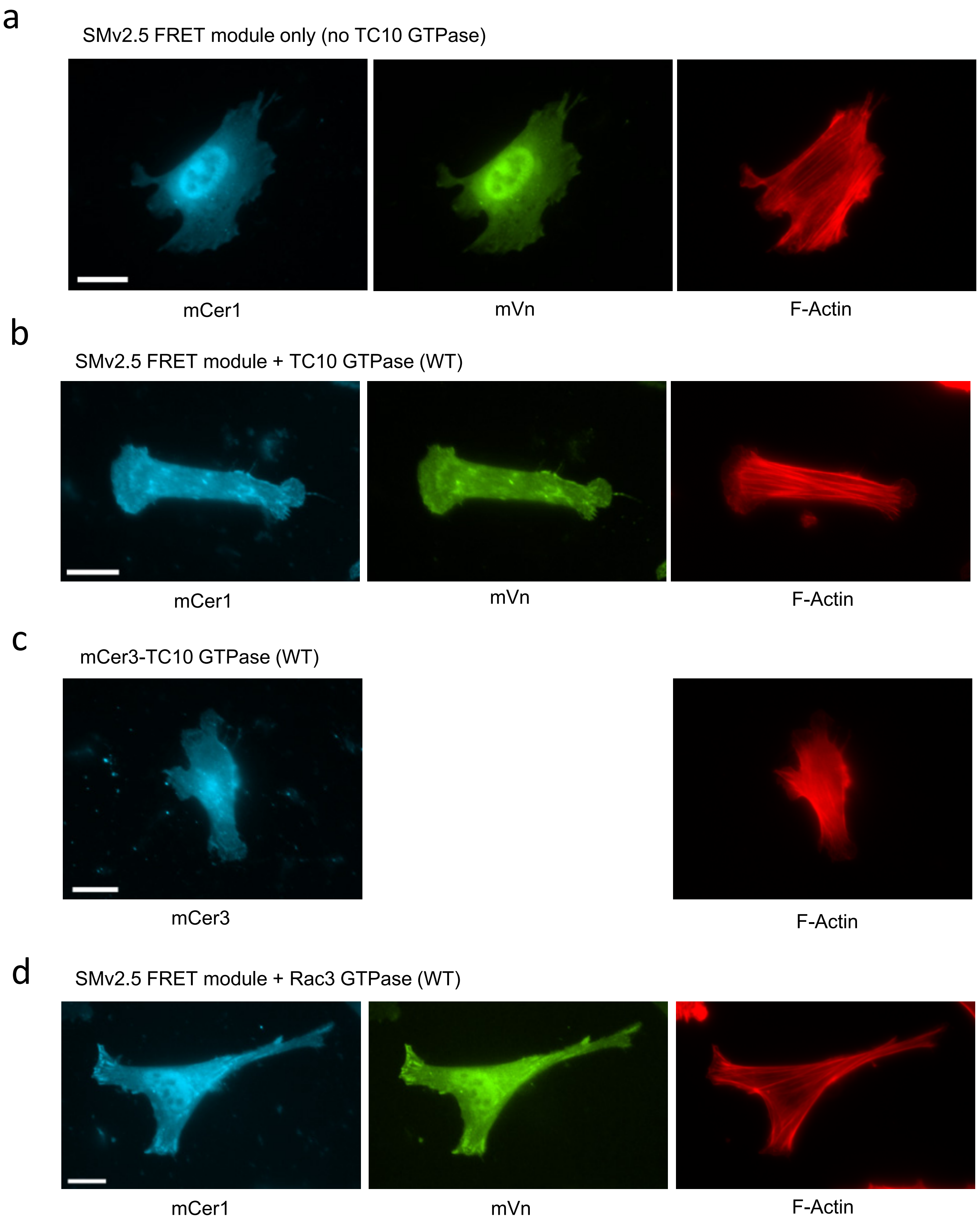

Supplementary Figure 9: Representative images of the comparisons of the FRET biosensor expression pattrens in mouse embryonic fibroblasts (MEF/3T3). **a.** Synonymously modified (SM) version 2.5 FRET detection module consisting of mCerulean1–tandem PBD domains–cp229mVenus. **b**. Same as (**a**) but a full length TC10-WT was attached at the C-terminus to make the TC10 FRET biosensor as described in this work. **c**. Expression of mCerulean3-TC10-WT. **d**. Same as (**a**) but a full length Rac3-WT was attached at the C-terminus to make the Rac3 FRET biosensor, as previously described (Donnelly, et al. 2017). White bar = 20μm. F-actin phalloidin counter stains are also shown.

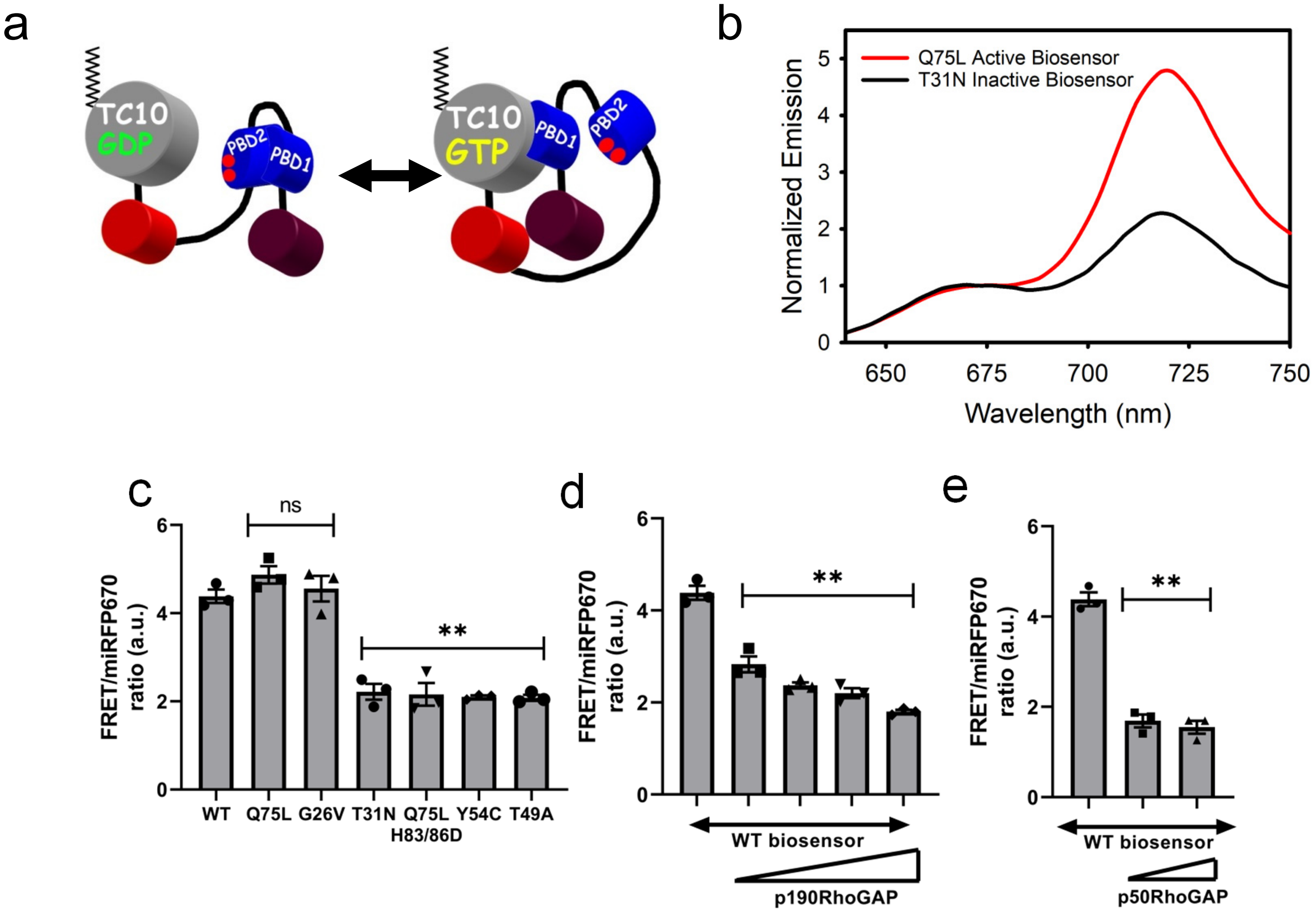

Supplementary Figure 10: TC10 FRET biosensor in near-infrared. **a.** FRET biosensor design using miRFP670 (shown as red) and miRFP720 (shown as purple) as the FRET pair. **b.** Representative, normalized fluorescence emission spectra, from 640 to 750 nm, upon 600 nm excitation. Spectra are normalized to emission at 670nm. **c.** Biosensor fluorometric characterization in HEK293T cells overexpressing the mutant versions of the biosensor. WT versus Q75L: p = 0.1219; WT versus G26V: p = 0.6240; WT versus T31N: p = 0.000764; WT versus Q75L-H83/86D: p = 0.001123; WT versus T49A: p = 0.0001550; WT versus Y54C: p = 0.0001240. N = 3 experiments, two-tailed Student's t-test. **d.** Biosensor response to p190RhoGAP titration in HEK293T cells. **p = 0.002521, 0.0002622, 0.0003001, and 0.00007985, respectively, for the GAP titration. N = 3 experiments, two-tailed Student's t-test. **e.** Biosensor response to p50RhoGAP titration in HEK293T cells. **p = 0.002019 and 0.0001703 for GAP titration. N = 3 experiments, two-tailed Student's t-test.

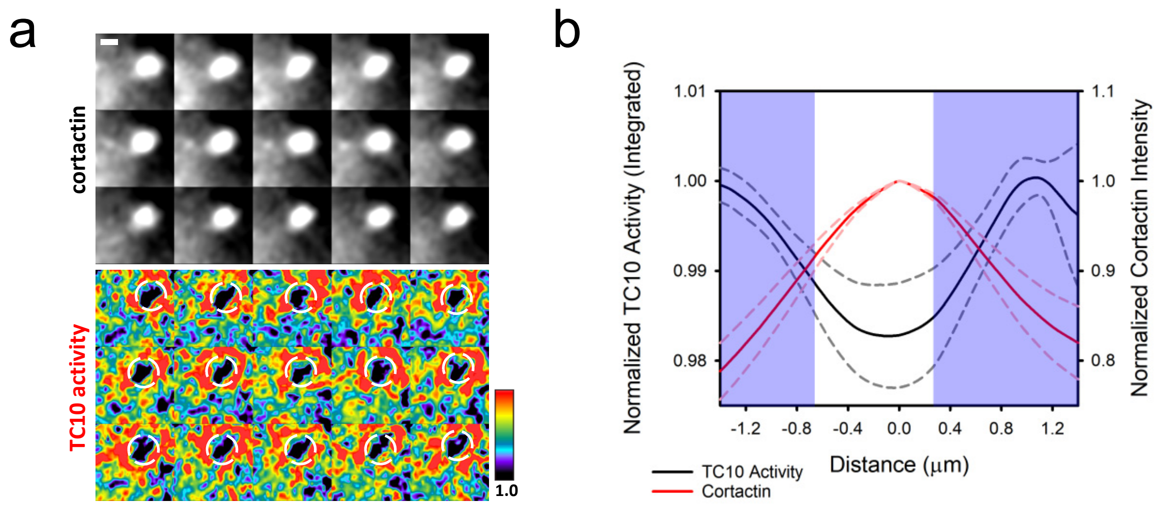

Supplementary Figure 11: TC10 FRET biosensor expressed in MDA-MB231 cells and imaged at an invadopodium. **a.** A representative time-lapse panel showing an invadopodium, denoted by cortactin staining, and the corresponding TC10 activity. The images within this panel were acquired at consecutive 10-second intervals. White bar = 0.75 μm. Pseudo-color scale for TC10 activity: 1.0 to 1.13, black to red. **b.** The line scan analysis of the intensity distributions across multiple invadopodia, showing normalized TC10 activity integrated over time and plotted against the matching normalized cortactin intensity distributions. The blue-shaded regions indicate significant ($p < 0.05$; one-tailed Student's t-test; $n = 12$ invadopodia from 7 cells over 4 experiments; p-value distributions are shown in Supplementary Data 1) differences compared with the TC10 activity intensity at the center of the invadopodia core, defined as 0.0 μm. Line scans were normalized to the local maxima of TC10 activity at the ring-like region surrounding the invadopodia core, which was denoted by the cortactin spot. The cortactin intensity was normalized at the center position, taken as the maximal intensity location along the line scans.

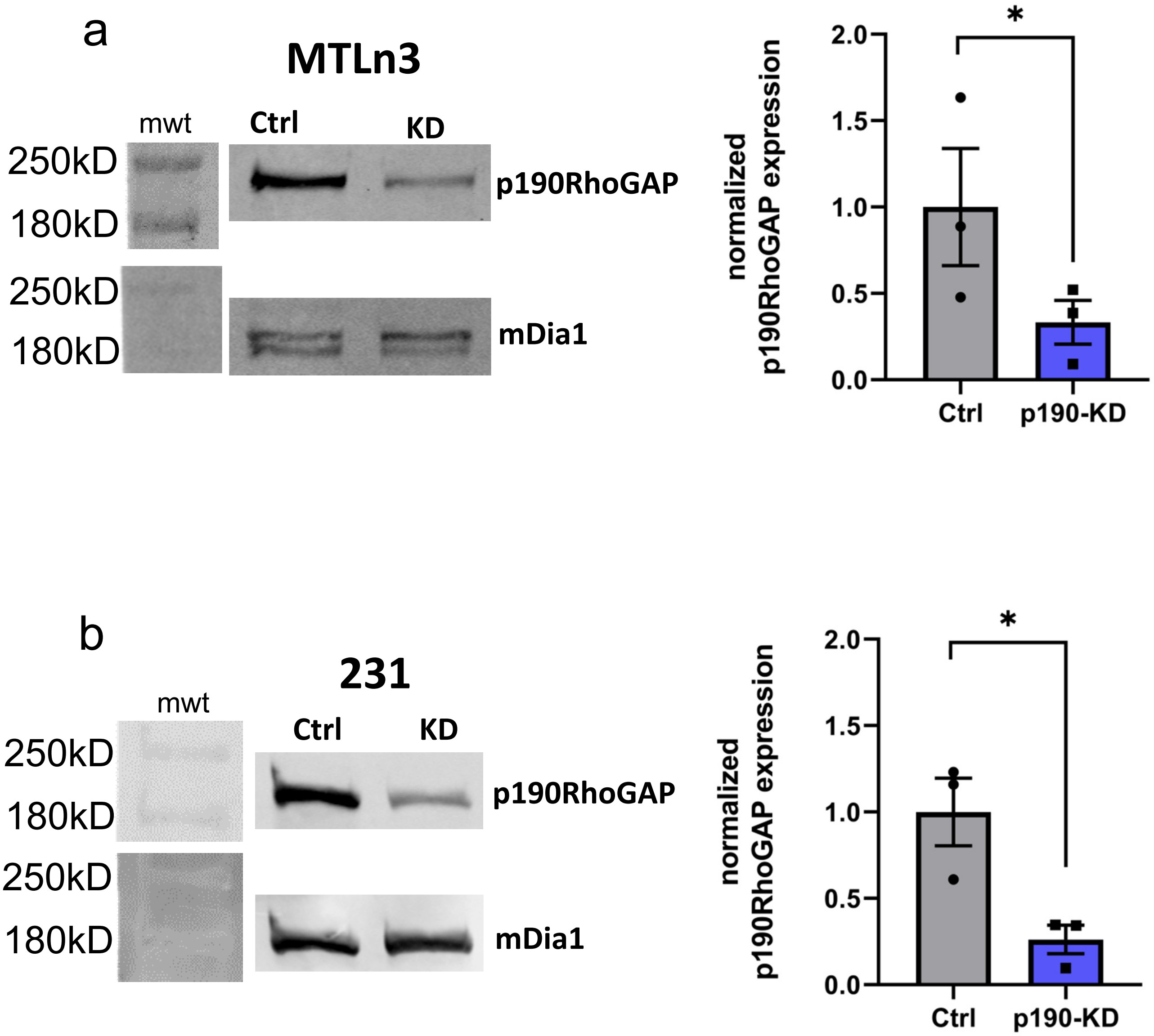

Supplementary Figure 12: p190RhoGAP depletion by siRNA. **a.** p190RhoGAP depletion analysis performed in MTLn3 cells. Representative western blot showing the expression levels of p190RhoGAP and the loading control. Quantification of p190RhoGAP depletion is also shown. $*p = 0.04883$. **b.** p190RhoGAP depletion analysis performed in MDA-MB231 cells. Representative western blot showing the expression levels of p190RhoGAP and the loading control. Quantification of p190RhoGAP depletion is also shown. $*p = 0.01164$. Paired, one-tailed Student's t-test was performed, n = 3 experiments, error bars represent the SEM. Full-sized western blots are shown in Supplementary Figure 19. KD, knockdown. All quantifications are normalized to Ctrl.

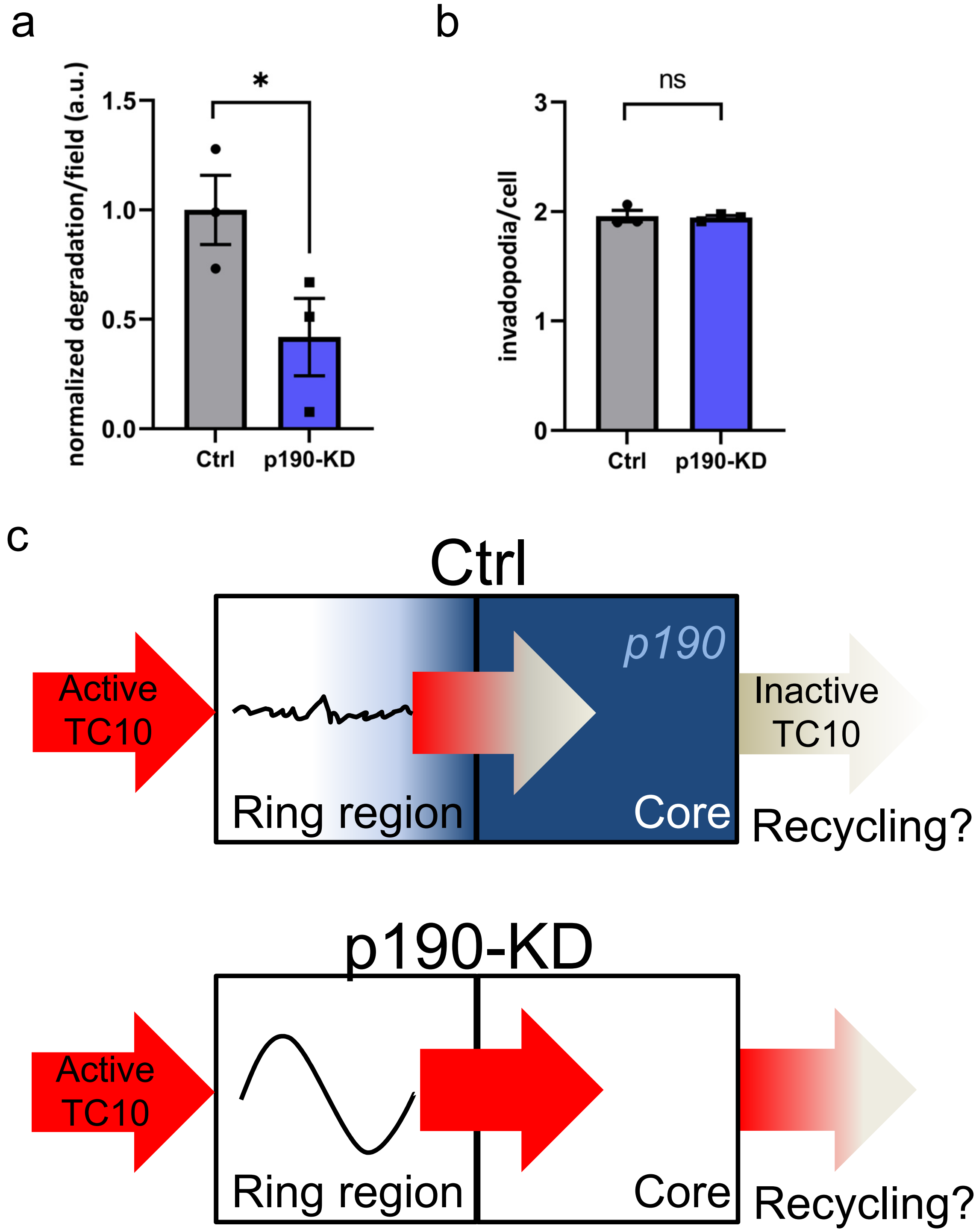

Supplementary Figure 13: Depletion of p190RhoGAP impacts matrix degradation but not invadopodia numbers in MDA-MB231 cells. **a.** p190RhoGAP depletion resulted in reduced matrix degradation at invadopodia. *p = 0.02469; one-tailed Student's t-test, n ≥ 50 invadopodia from ≥ 25 cells for each condition, from 3 experiments. **b.** p190RhoGAP depletion did not impact the steady-state invadopodia numbers. "ns", not significant; p = 0.4268; two-tailed Student's t-test, n ≥ 50 invadopodia from ≥ 25 cells for each condition, from 3 experiments. **c.** A schematic depiction of TC10 activity flux through the ring-like region surrounding invadopodia core and the core of invadopodia. The active TC10 is depicted in red (does not correspond to the bulk "amount" of TC10 but to the activity status of TC10), p190RhoGAP in blue. Depetion of p190RhoGAP eliminates the "reaction" pathway in a traditional mass-balance depiction shown here, reducing the system to that driven only by the flux. This model suggests a stochastic recycling pathway resulting in random fluctuations in TC10 activity observed in the core of invadopodia.

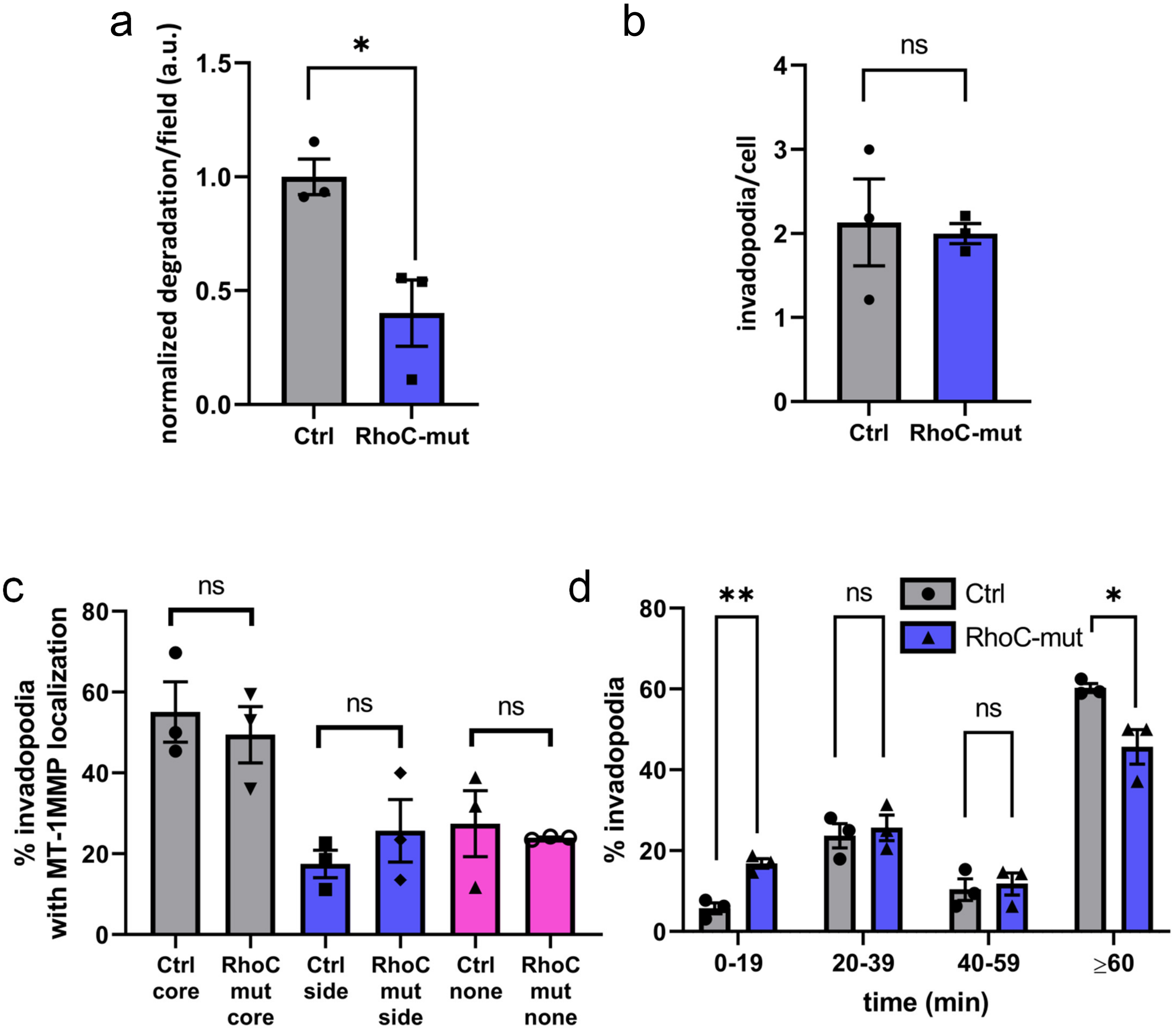

Supplementary Figure 14: Activated mutant of RhoC impacts invadopodia functions. **a.** Overexpression of RhoC F30L/E93H/N94H mutant (RhoC-mut) impacted matrix degradation in MTLn3 cells. *p = 0.01765; one-tailed Student's t-test, n=3 experiments. **b.** Overexpression of RhoC-mut did not impact the steady-state number of invadopodia in MTLn3 cells. "ns", not significant, p = 0.8022; two-tailed Student's t-test, n=3 experiments. Quantifications in (**a**) is normalized against the Ctrl. **c.** Localization of MT1-MMP at invadopodia did not changed when activated but GAP-binding deficient RhoC was overexpressed. "ns", not significant. Core, p = 0.4869; side, p = 0.5160; none p=0.7152; paired, two-tailed Student's t-test, n=3 experiments. **d.** Invadopodia lifetimes in MTLn3 cells, shown as a histogram with bins corresponding to 20-min intervals. **p = 0.003343; *p = 0.03022; two-tailed Student's t-test. For all experiments, n ≥ 50 invadopodia from ≥ 25 cells for each condition, from 3 experiments.

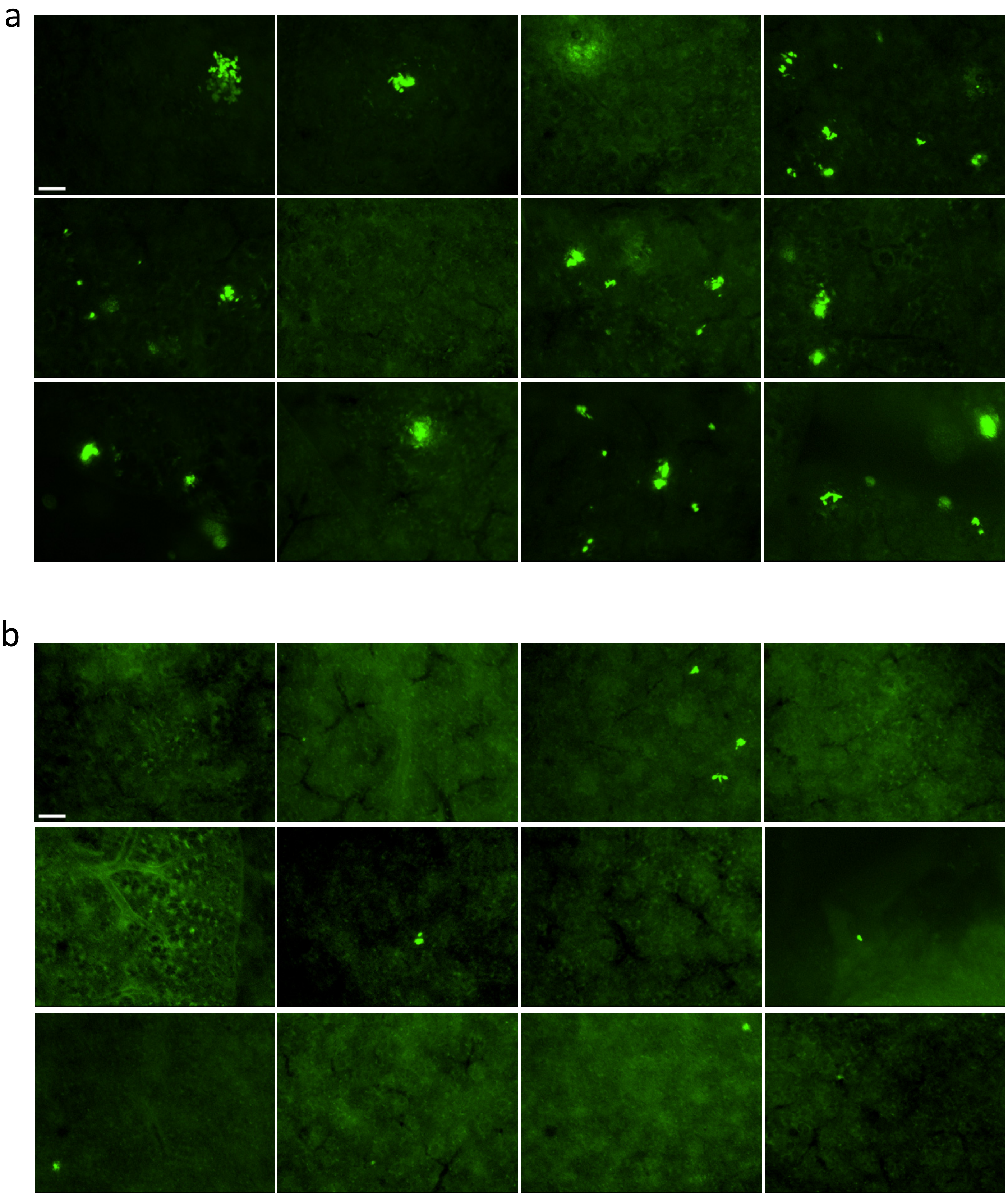

Supplementary Figure 15: Additional examples of the fields of view of mouse lung metastasis. **a**. NT-Ctrl MTLn3 lung metastasis, random fields of view at 20x magnification. **b**. TC10-KO MTLn3 lung metastasis, random fields of view at 20x magnification. White bar = 100μm.

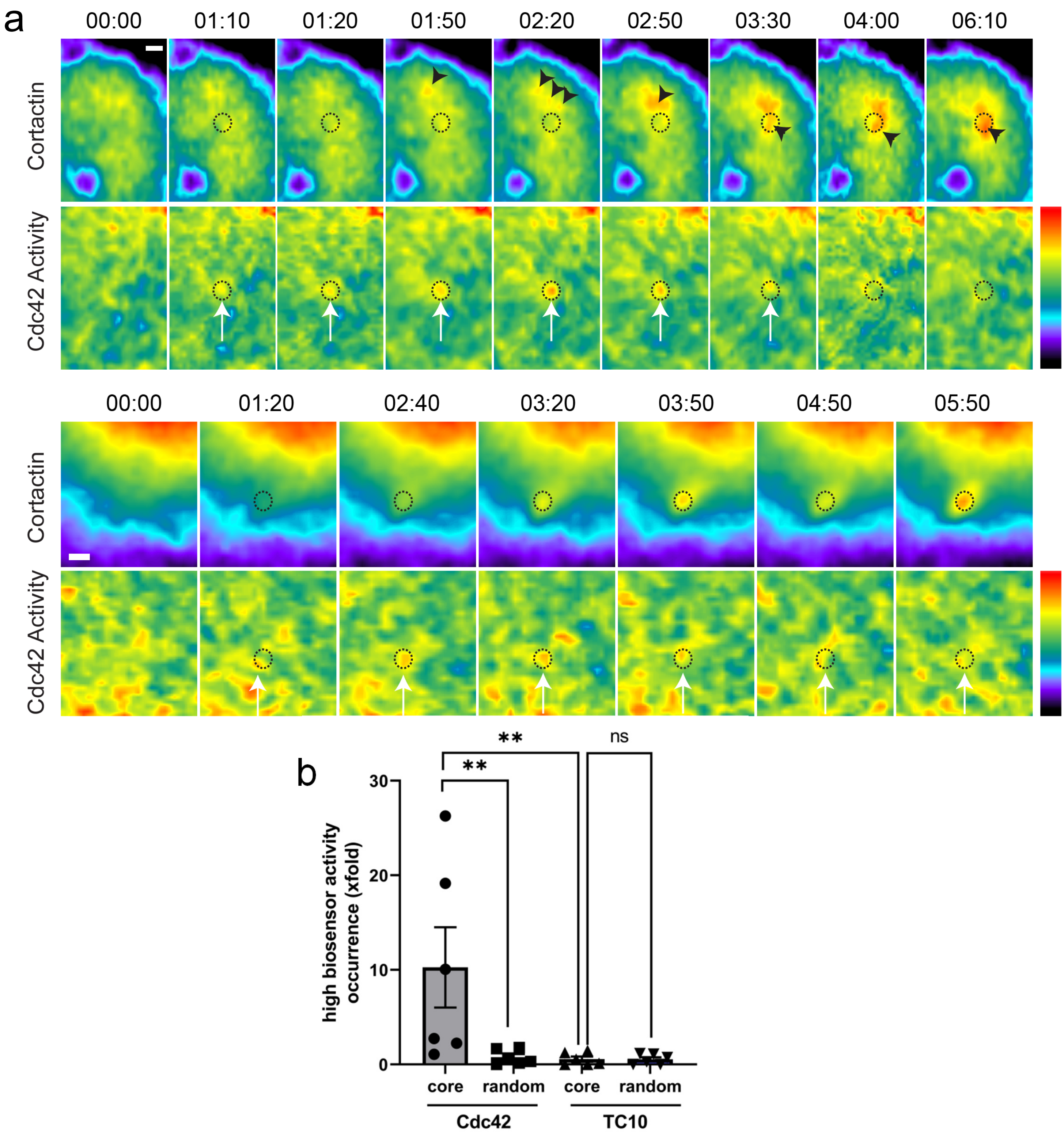

Supplementary Figure 16: Cdc42 biosensor activity at invadopodia precursors in MDA-MB231 cells. **a.** Representative time-lapse panels showing Cdc42 activity during invadopodia precursor formation in MDA-MB231 cells. White bar = 1 μm. (Top): pseudo-color scale for Cdc42 biosensor images corresponds to 1.0 (black) to 1.29 (red). (Bottom): pseudo-color scale for Cdc42 biosensor images corresponds to 1.0 (black) to 1.36 (red). Cortactin channels are also pseudo-colored to increase the contrast. White arrows indicate Cdc42 activity within the Core of a nascent invadopodium. Black arrowheads show the region of cortactin appearance and the coalescence into an invadopodium core. **b.** Ratio of the occurrence of high biosensor activity (>+1.0 SD from the mean) within the core of invadopodium during formation to the background activity levels in MDA-MB231 cells. **p = 0.00657 for Cdc42 activity in the invadopodium core versus a random background location; **p = 0.00657 for Cdc42 versus TC10 activities in the invadopodium core; = 0.46812 for TC10 activity in the invadopodium core versus Rnd. One-tailed Mann-Whitney U test, n = 6 invadopodia from 5 cells, from 3 experiments.

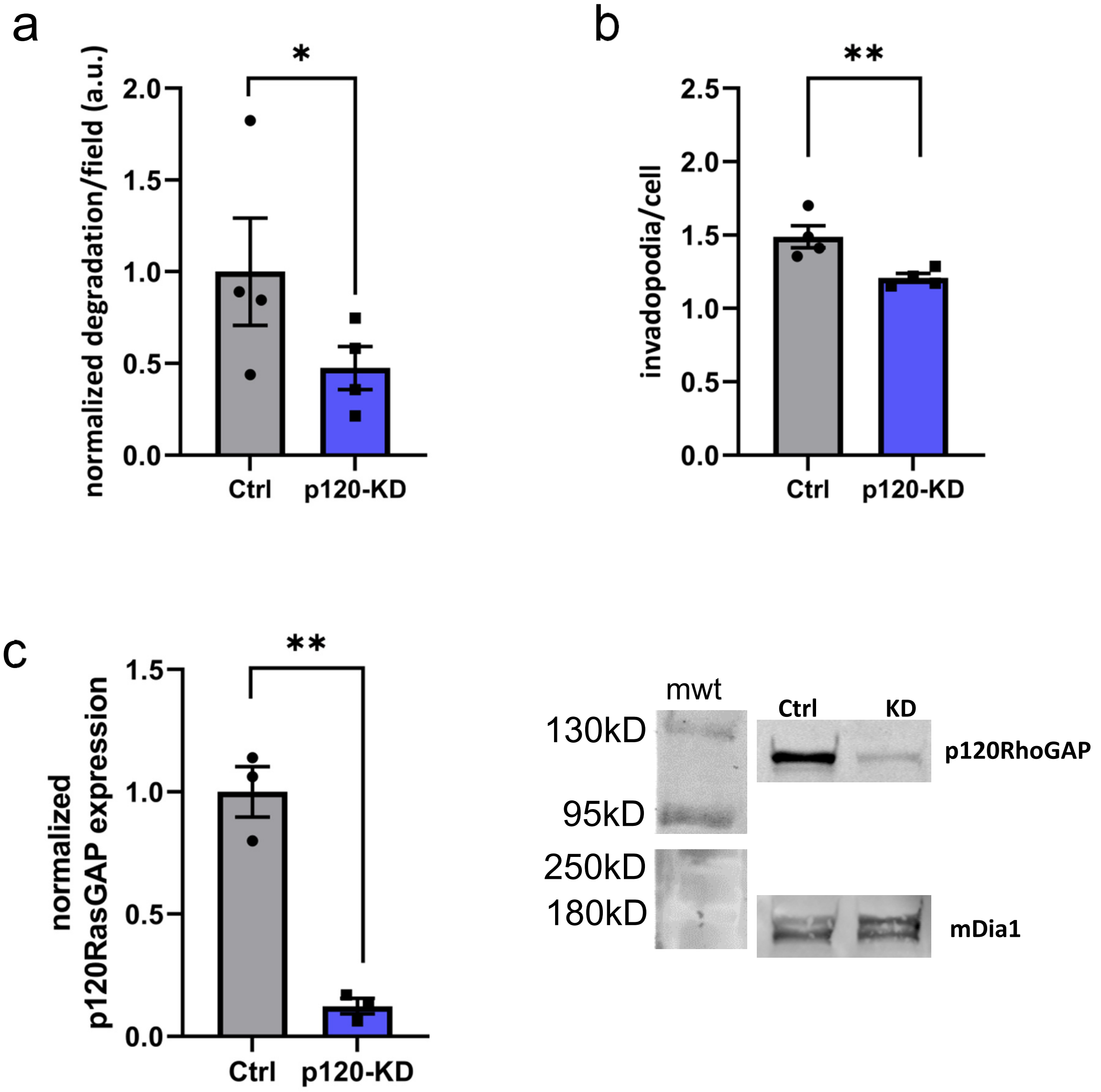

Supplementary Figure 17: Depletion of p120RasGAP impacts invadopodia functions. **a.** p120RasGAP depletion modestly impacts matrix degradation in MTLn3 cells. *p = 0.04962, paired, one-tailed Student's t-test. **b.** p120RasGAP depletion impacts the steady-state number of invadopodia in MTLn3 cells. **p = 0.004434, paired, one-tailed Student's t-test. **c.** p120RasGAP depletion efficiency and representative western blots. **p = 0.0075, one-tailed Student's t-test. In (**a**) and (**b**), n ≥ 50 invadopodia from ≥ 25 cells for each condition, from 3 experiments. In (**c**), n = 3 experiments. Quantifications are normalized against Ctrl. Full-sized western blots are shown in Supplementary Figure 19.

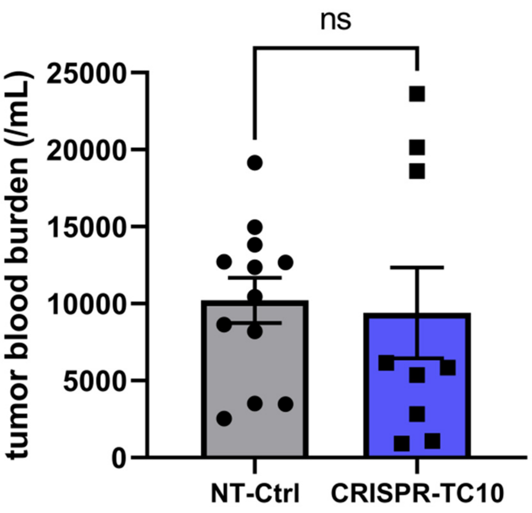

Supplementary Figure 18: Circulating tumor cell counts *in vivo* are not different in mice bearing the CRISPR-TC10-depleted tumor compared to the control mice bearing the non-targeting CRISPR/cas9 tumor. Ns p = 0.7936, two-tailed Student's t-test, n = 12 for NT-Ctrl mice and 9 for CRISPR-TC10 mice.

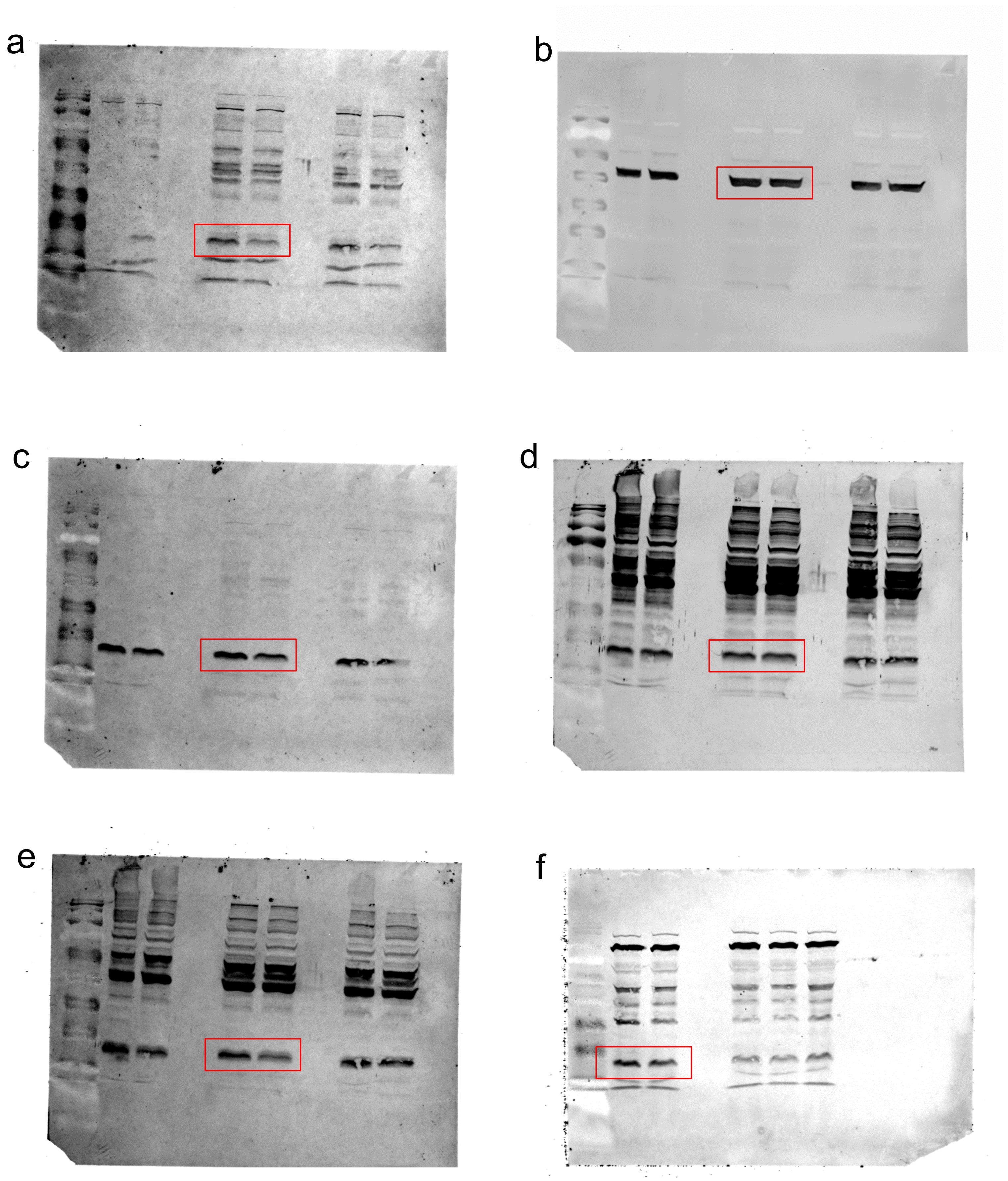
a
b
c
d
e
f

g

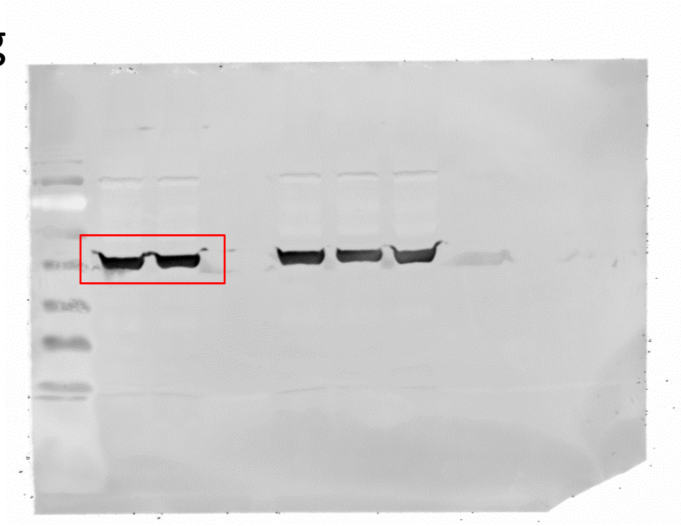

h

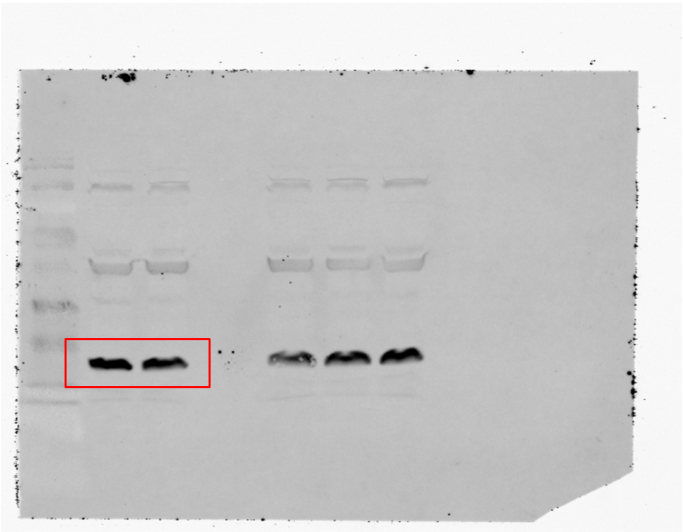

i

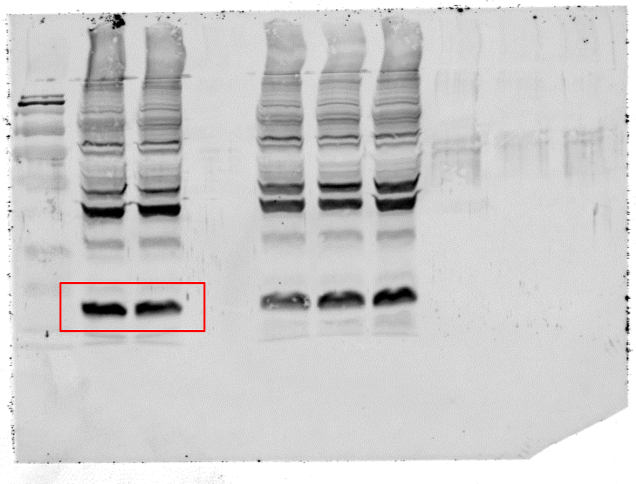

j

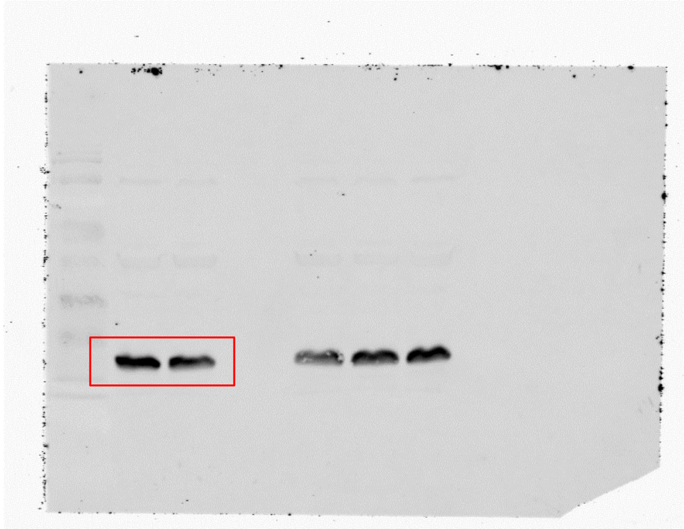

k

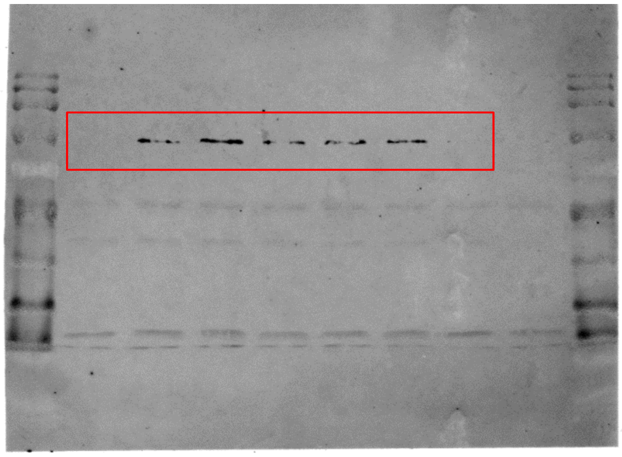

l

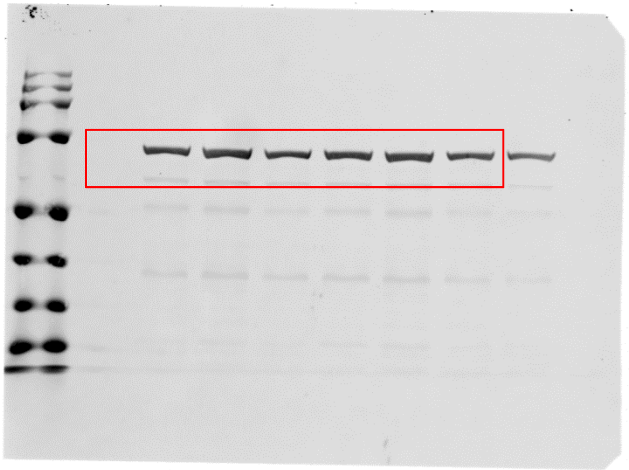

m

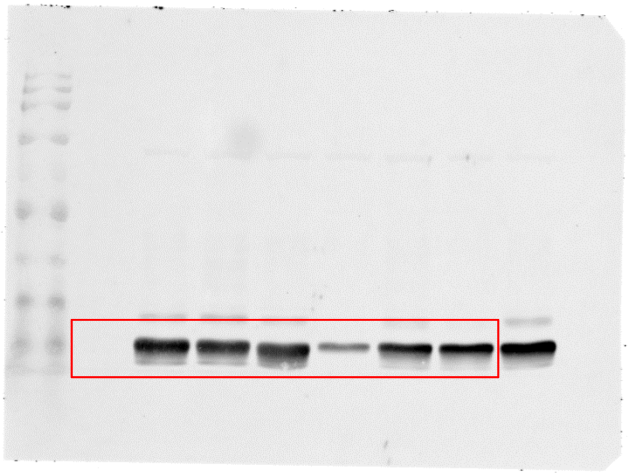

n

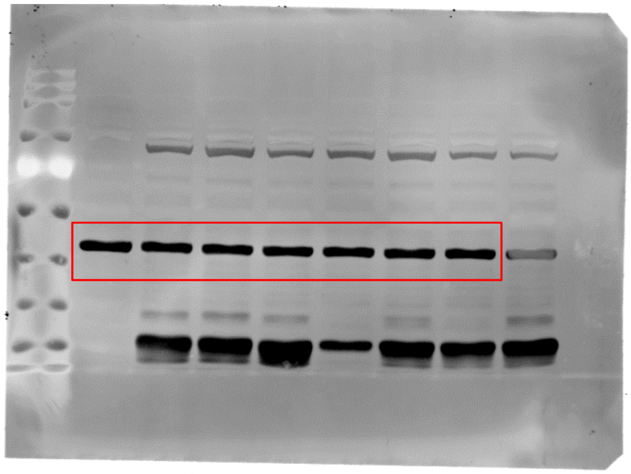

o

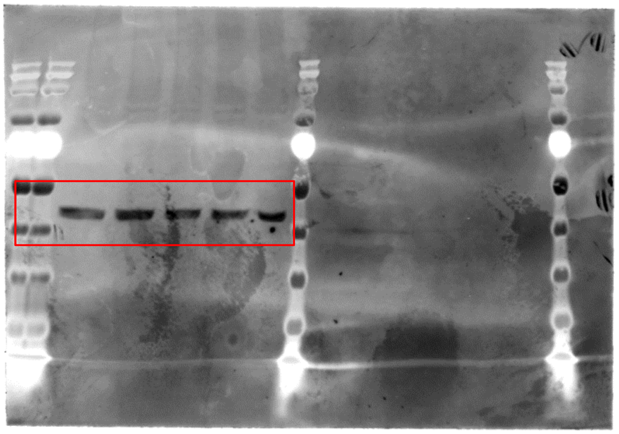

p

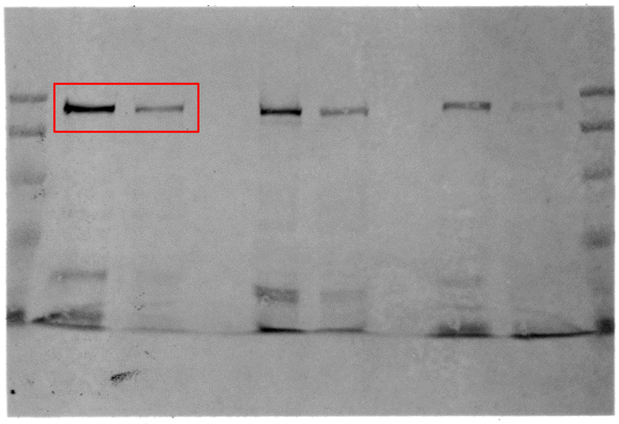

q

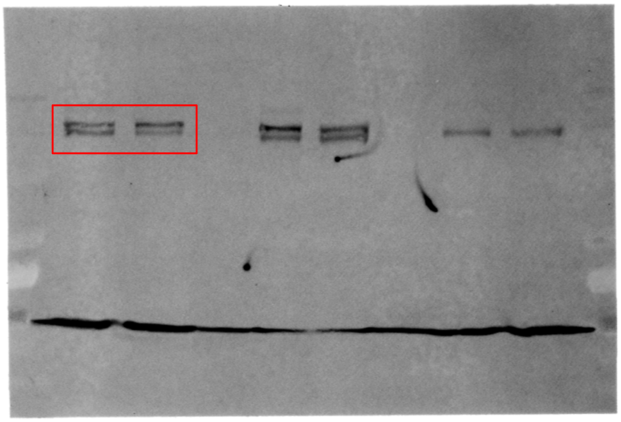

r

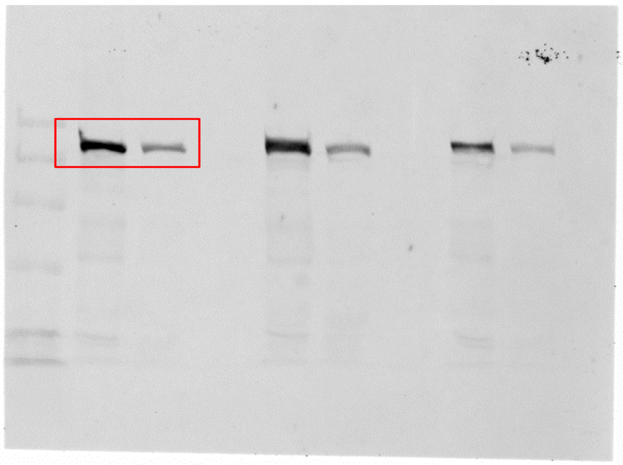

s

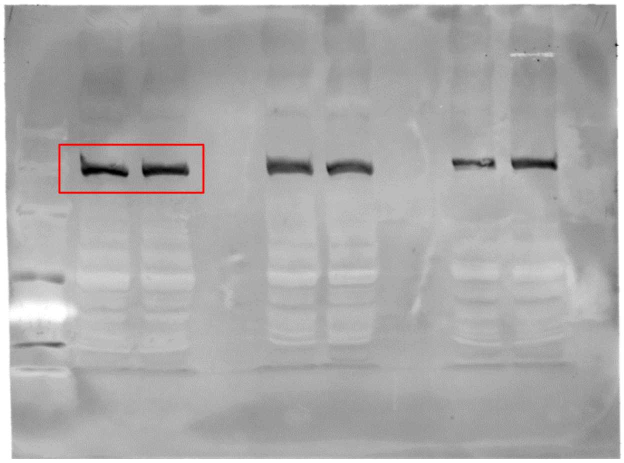

t

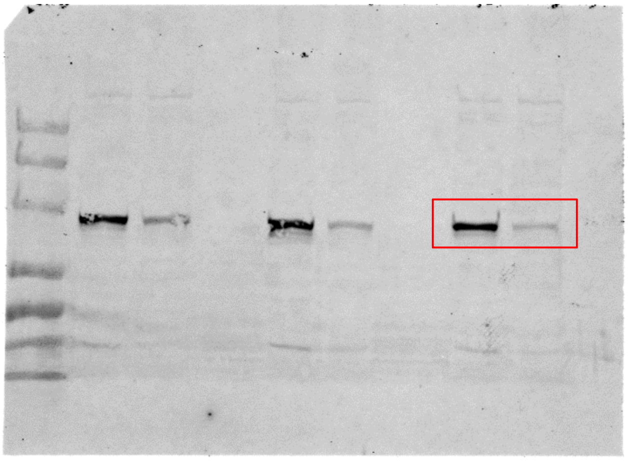

u

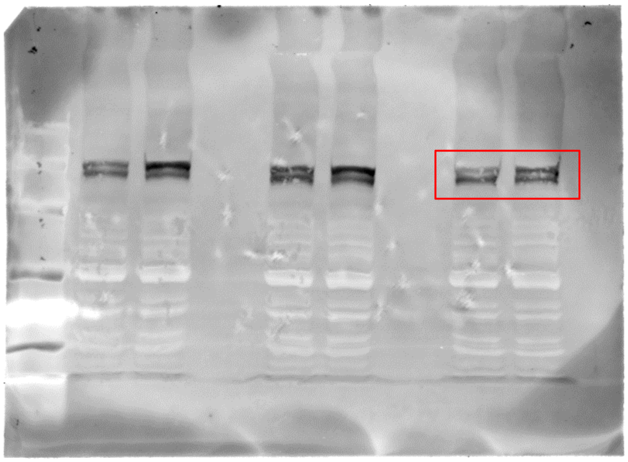

v

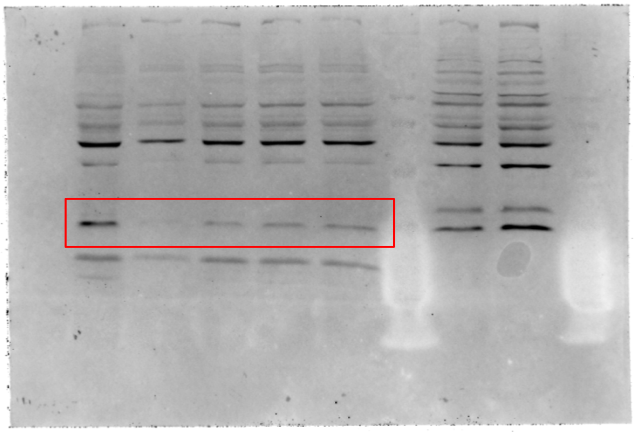

w

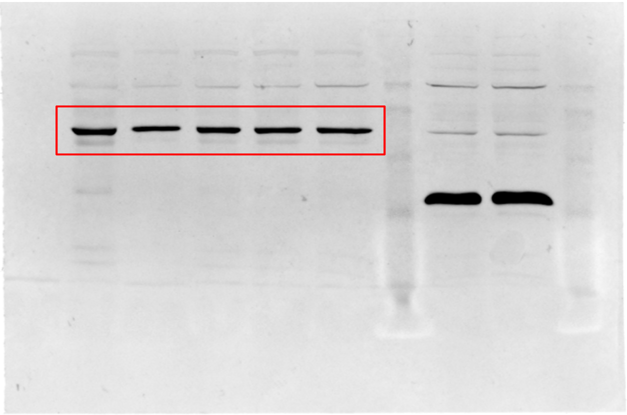

x

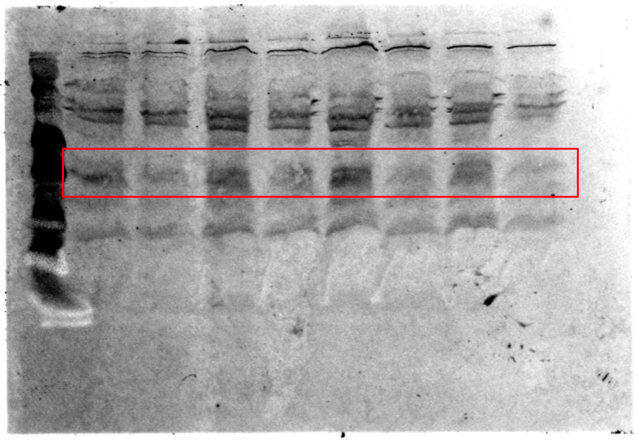

y

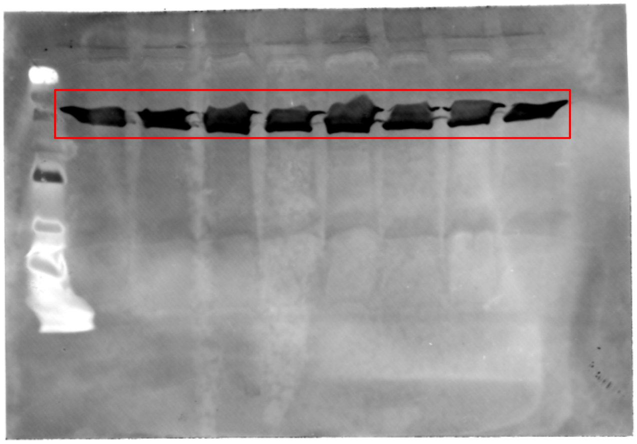

z

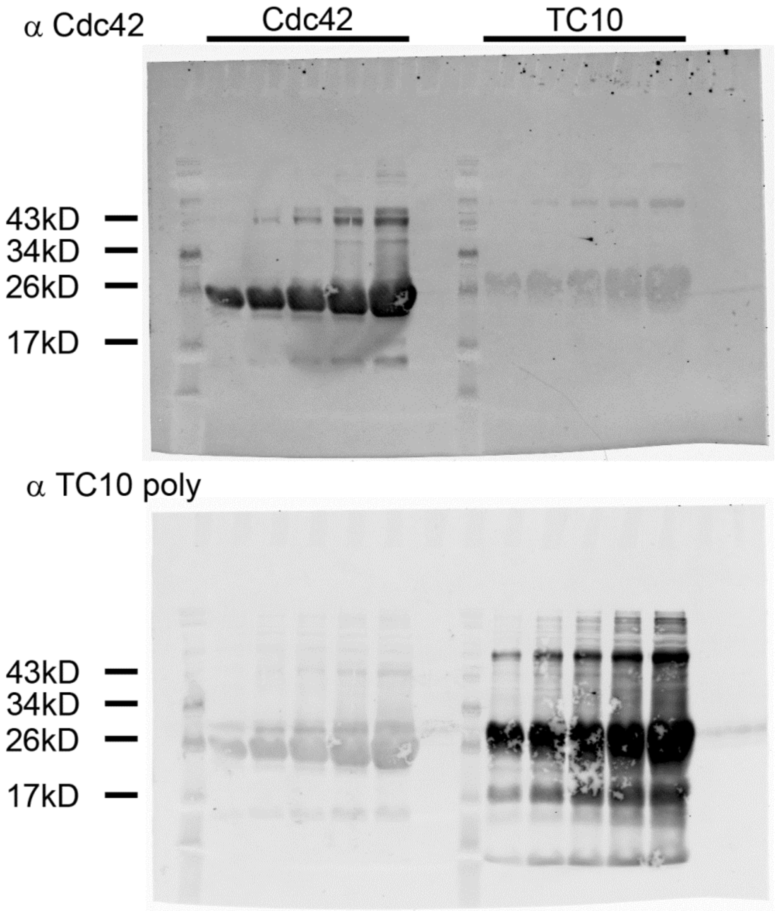

Supplementary Figure 19: Uncropped western blot images. **a.** Supplementary Figure 3, detecting TC10. **b.** Supplementary Figure 3, detecting β-Actin. **c.** Supplementary Figure 3, detecting Cdc42. **d.** Supplementary Figure 3, detecting Rac1. **e.** Supplementary Figure 3, detecting RhoA. **f.** Supplementary Figure 4, detecting TC10. **g.** Supplementary Figure 4, detecting β-Actin. **h.** Supplementary Figure 4, detecting Cdc42. **i.** Supplementary Figure 4, detecting Rac1. **j.** Supplementary Figure 4, detecting RhoA. **k.** Figure 3k, detecting MYC. **l.** Figure 3k, detecting MYC. **m.** Figure 3k, detecting FLAG. **n.** Figure 3k, detecting β-Actin. **o.** Supplementary Figure 8a, detecting β-Actin. **p.** Supplementary Figure 12a, detecting p190RhoGAP-A. **q.** Supplementary Figure 12a, detecting mDia1. **r.** Supplementary Figure 12b, detecting p190RhoGAP-A. **s.** Supplementary Figure 12b, detecting mDia1. **t.** Supplementary Figure 17c, detecting p120RasGAP. **u.** Supplementary Figure 17c, detecting mDia1. **v.** Figure 7c, detecting TC10. **w.** Figure 7c, detecting β-Actin. **x**. Figure 7d, detecting TC10. **y**. Figure 7d, detecting β-Actin. **z**. Confirmation of antibody specificities between Cdc42 (Santa Cruz Biotechnology mouse monoclonal: clone B-8, sc-8401) and TC10 (Novus rabbit polyclonal: 07-2151), using purified human Cdc42 and TC10, loaded in increasing amounts in each well.
