# Supplementary material for: TC10 regulates breast cancer invasion and metastasis by controlling membrane type-1 matrix metalloproteinase at invadopodia
Source: Commun Biol. 2021 Sep 16;4:1091. doi: 10.1038/s42003-021-02583-3 (PMC8445963; doi:10.1038/s42003-021-02583-3)
Supplement: Supplementary file 2 — Description of Supplementary Files [file 42003_2021_2583_MOESM2_ESM.pdf]

Description of Additional Supplementary Files

File name: Supplementary Movie 1

Description: mNeonGreen-TC10 WT expressed together with mScarlet-Cortactin in MTLn3 cells, imaged at 10s intervals at 60x magnification with TIRF excitation penetration depth of 360nm from the coverslip. Frame playback rate: 12 fps, white bar = 2-µm.

File name: Supplementary Movie 2

Description: mNeonGreen-TC10 WT expressed together with mtagBFP2-Cortactin and MT1-MMP-mCherry in MTLn3 cells, imaged at 10s intervals at 60x magnification with TIRF excitation penetration depth of 360nm from the coverslip. Frame playback rate: 10 fps, white bar = 5-µm.

File name: Supplementary Movie 3

Description: mNeonGreen-TC10 WT expressed together with mtagBFP2-Cortactin and MT1-MMP-mCherry in MTLn3 cells, imaged at 10s intervals at 60x magnification with TIRF excitation penetration depth of 360nm from the coverslip. Frame playback rate: 7 fps, white bar = 2-µm.

File name: Supplementary Movie 4

Description: FRET biosensor for TC10 expressed together with miRFP703-Cortactin, treated with Ctrl siRNA, imaged at 10s intervals at 60x magnification in widefield epifluorescence excitation. Frame playback rate: 5 fps, white bar = 1-µm.

File name: Supplementary Movie 5

Description: FRET biosensor for TC10 expressed together with miRFP703-Cortactin, treated with siRNA against p190RhoGAP, imaged at 10s intervals at 60x magnification in widefield epifluorescence excitation. Frame playback rate: 5 fps, white bar = 1-µm.

File name: Supplementary Data 1

Description: Student t-test p-value limits for Linescan analyses of MTLn3 cell invadopodia. Details of the statistical analyses can be found in the figure legends for the corresponding linescan plots in the main Figure panels. Red-highlighted numbers show $p<0.05$ compared to the center of invadopodia, as determined by the position of cortactin intensity maxima.

File name: Supplementary Data 2

Description: Theoretical base sequences of the cyan-yellow FRET and the NIR FRET biosensors for TC10 GTPase.

File name: Supplementary Data 3

Description: The source data used to plot the graphs in the main panel figures.
