# Supplementary material for: TC10 regulates breast cancer invasion and metastasis by controlling membrane type-1 matrix metalloproteinase at invadopodia
Source: Commun Biol. 2021 Sep 16;4:1091. doi: 10.1038/s42003-021-02583-3 (PMC8445963; doi:10.1038/s42003-021-02583-3)
Supplement: Supplementary file 8 — Supplementary Data 1 [file 42003_2021_2583_MOESM8_ESM.pdf]

Supplementary Data 1:

P-value limits for Linescan analyses of invadopodia

Figure 4I: MTLn3 invadopodia

Red-highlighted cells show p<0.05 compared to the center of invadopodia

X-position (micron)	p-value
-2.325	0.333324342
-2.2875	0.208341653
-2.25	0.296269208
-2.2125	0.350796313
-2.175	0.284425359
-2.1375	0.101854135
-2.1	0.039761836
-2.0625	0.020733754
-2.025	0.440452375
-1.9875	0.475158242
-1.95	0.472519412
-1.9125	0.446133117
-1.875	0.380729702
-1.8375	0.316636021
-1.8	0.254786302
-1.7625	0.19000394
-1.725	0.125409147
-1.6875	0.417552361
-1.65	0.276192979
-1.6125	0.128916081
-1.575	0.032170484
-1.5375	0.009330844
-1.5	0.007353831
-1.4625	0.006621893
-1.425	0.005689452
-1.3875	0.005328448
-1.35	0.005049157
-1.3125	0.037907498
-1.275	0.014168476
-1.2375	0.006406293
-1.2	0.004126149
-1.1625	0.003475415
-1.125	0.003334697

-1.0875	0.003172104
-1.05	0.002818788
-1.0125	0.002572025
-0.975	0.002255678
-0.9375	0.002085959
-0.9	0.002029547
-0.8625	0.002112927
-0.825	0.002409877
-0.7875	0.003175228
-0.75	0.004607168
-0.7125	0.00695018
-0.675	0.010806163
-0.6375	0.016747789
-0.6	0.026183937
-0.5625	0.03979324
-0.525	0.058705123
-0.4875	0.079897876
-0.45	0.108509542
-0.4125	0.14119996
-0.375	0.187114707
-0.3375	0.236470397
-0.3	0.288371794
-0.2625	0.332177156
-0.225	0.375859459
-0.1875	0.407927339
-0.15	0.416348955
-0.1125	0.439032497
-0.075	0.499316663
-0.0375	0.456031005
0	
0.0375	0.396179108
0.075	0.342177935
0.1125	0.305097247
0.15	0.32503061
0.1875	0.316373871
0.225	0.307210677
0.2625	0.287808159
0.3	0.266467949
0.3375	0.235618967
0.375	0.215571055
0.4125	0.19078104
0.45	0.158410646
0.4875	0.126241655

0.525	0.101024423
0.5625	0.082839771
0.6	0.068551121
0.6375	0.0583591
0.675	0.054496712
0.7125	0.050498075
0.75	0.047386757
0.7875	0.043311871
0.825	0.04074987
0.8625	0.038905284
0.9	0.036345277
0.9375	0.03611919
0.975	0.035589072
1.0125	0.033285296
1.05	0.031821307
1.0875	0.030761876
1.125	0.030350513
1.1625	0.030398241
1.2	0.031320882
1.2375	0.032505294
1.275	0.034634523
1.3125	0.037962288
1.35	0.041484272
1.3875	0.045362593
1.425	0.048703985
1.4625	0.05276189
1.5	0.055798638
1.5375	0.058677506
1.575	0.059607995
1.6125	0.059010065
1.65	0.048689868
1.6875	0.047343311
1.725	0.051247473
1.7625	0.048087206
1.8	0.046222397
1.8375	0.043829358
1.875	0.043272947
1.9125	0.041425821
1.95	0.038361493
1.9875	0.047915285
2.025	0.044578962
2.0625	0.06020391
2.1	0.039472482

2.1375	0.036986344
2.175	0.033465416
2.2125	0.025079735
2.25	0.0176303
2.2875	0.058425873
2.325	0.186300019
2.3625	0.110356492
2.4	0.092503273
2.4375	0.268574024
2.475	0.184732528

Figure 5h: MTLn3 invadopodia p190-KD

Red-highlighted cells show p<0.05 compared to the center of invadopodia

X-position (micron)	p-value
-2.25	0.050782474
-2.2125	0.075852293
-2.175	0.058310925
-2.1375	0.069475634
-2.1	0.103179033
-2.0625	0.063463629
-2.025	0.058986632
-1.9875	0.060570371
-1.95	0.053611941
-1.9125	0.052763864
-1.875	0.05209951
-1.8375	0.053737485
-1.8	0.050279228
-1.7625	0.047196872
-1.725	0.044118186
-1.6875	0.041878844
-1.65	0.039594551
-1.6125	0.037208705
-1.575	0.034750065
-1.5375	0.032906844
-1.5	0.03180472
-1.4625	0.03099728
-1.425	0.031654407
-1.3875	0.031302972
-1.35	0.030267969

-1.3125	0.029111931
-1.275	0.027360577
-1.2375	0.025886194
-1.2	0.024851269
-1.1625	0.024331694
-1.125	0.02421346
-1.0875	0.024118902
-1.05	0.024594397
-1.0125	0.025593928
-0.975	0.026312657
-0.9375	0.025785745
-0.9	0.024671493
-0.8625	0.024796326
-0.825	0.025606237
-0.7875	0.025868018
-0.75	0.026771134
-0.7125	0.028048212
-0.675	0.029914769
-0.6375	0.032141399
-0.6	0.036277966
-0.5625	0.041224043
-0.525	0.050654095
-0.4875	0.061725638
-0.45	0.079982369
-0.4125	0.104205557
-0.375	0.135419574
-0.3375	0.177505206
-0.3	0.212942247
-0.2625	0.225119507
-0.225	0.235656429
-0.1875	0.252797892
-0.15	0.29076287
-0.1125	0.324339139
-0.075	0.348726193
-0.0375	0.48153016
**0**	
0.0375	0.013424108
0.075	0.016465449
0.1125	0.028289859
0.15	0.039791478
0.1875	0.049857137
0.225	0.055551281

0.2625	0.057721995
0.3	0.063805393
0.3375	0.063415755
0.375	0.065701646
0.4125	0.06661752
0.45	0.064690615
0.4875	0.064528098
0.525	0.067004869
0.5625	0.070253298
0.6	0.074282161
0.6375	0.075676377
0.675	0.076436752
0.7125	0.074520357
0.75	0.074045691
0.7875	0.072314384
0.825	0.07144668
0.8625	0.070543247
0.9	0.067955492
0.9375	0.064131489
0.975	0.059360173
1.0125	0.055602025
1.05	0.051282496
1.0875	0.047167878
1.125	0.044488198
1.1625	0.041270876
1.2	0.035261731
1.2375	0.034197906
1.275	0.031022597
1.3125	0.028537317
1.35	0.026890378
1.3875	0.025598446
1.425	0.02446709
1.4625	0.023140167
1.5	0.021841825
1.5375	0.020486301
1.575	0.020292167
1.6125	0.020986661
1.65	0.022520364
1.6875	0.025722371
1.725	0.027792642
1.7625	0.029648492
1.8	0.030264515
1.8375	0.032114313

1.875	0.033724173
1.9125	0.036140348
1.95	0.03765679
1.9875	0.034573558
2.025	0.035654023
2.0625	0.039355849
2.1	0.033188557
2.1375	0.027523077
2.175	0.035337232
2.2125	0.0373626
2.25	0.037970843
2.2875	0.052986975
2.325	0.050337147
2.3625	0.057073567
2.4	0.218757277
2.4375	0.22532759
2.475	0.4880494
2.5125	0.455204192
2.55	0.403096376

Supplementary Figure 11b: MDA-MB-231 invadopodia

Red-highlighted cells show p<0.05 compared to the center of invadopodia

X-position (micron)	p-value
-2.4	0.36661081
-2.3625	0.27616192
-2.325	0.39031267
-2.2875	0.19272857
-2.25	0.10117501
-2.2125	0.02930602
-2.175	0.33271778
-2.1375	0.337406
-2.1	0.34443526
-2.0625	0.36192108
-2.025	0.12687112
-1.9875	0.03606141
-1.95	0.02423647
-1.9125	0.02708208
-1.875	0.02623878
-1.8375	0.02259117
-1.8	0.01958408
-1.7625	0.01691168

-1.725	0.01462997
-1.6875	0.01265248
-1.65	0.0110351
-1.6125	0.0099337
-1.575	0.00968663
-1.5375	0.0097351
-1.5	0.01024929
-1.4625	0.01114021
-1.425	0.01238654
-1.3875	0.01409839
-1.35	0.01585096
-1.3125	0.01771973
-1.275	0.01986259
-1.2375	0.02191907
-1.2	0.02345927
-1.1625	0.02493678
-1.125	0.02635447
-1.0875	0.02766339
-1.05	0.02890687
-1.0125	0.03019102
-0.975	0.03180102
-0.9375	0.03334727
-0.9	0.03522549
-0.8625	0.03740731
-0.825	0.03972603
-0.7875	0.04221071
-0.75	0.04497575
-0.7125	0.04913846
-0.675	0.05440852
-0.6375	0.06245967
-0.6	0.07170322
-0.5625	0.08608786
-0.525	0.10246767
-0.4875	0.12522983
-0.45	0.15485995
-0.4125	0.19483684
-0.375	0.23944077
-0.3375	0.29365732
-0.3	0.34009279
-0.2625	0.3861762
-0.225	0.43252048
-0.1875	0.48018583

-0.15	0.48317933
-0.1125	0.41352652
-0.075	0.37485747
-0.0375	0.31663943
**0**	
0.0375	0.11653715
0.075	0.09384527
0.1125	0.07028582
0.15	0.06634571
0.1875	0.05389081
0.225	0.04626272
0.2625	0.04161859
0.3	0.03711621
0.3375	0.03239859
0.375	0.02839538
0.4125	0.02566925
0.45	0.02422039
0.4875	0.02287208
0.525	0.02184326
0.5625	0.02105992
0.6	0.02048602
0.6375	0.02009409
0.675	0.01948284
0.7125	0.01889112
0.75	0.01815053
0.7875	0.0170939
0.825	0.01584866
0.8625	0.01470911
0.9	0.01328461
0.9375	0.01190243
0.975	0.01035734
1.0125	0.00861576
1.05	0.00711624
1.0875	0.00579983
1.125	0.00469465
1.1625	0.00400749
1.2	0.00401325
1.2375	0.00500566
1.275	0.00781752
1.3125	0.01406827
1.35	0.02440962
1.3875	0.03793254

1.425	0.05425427
1.4625	0.07294114
1.5	0.09541913
1.5375	0.12078098
1.575	0.15174364
1.6125	0.18427059
1.65	0.21690215
1.6875	0.24167521
1.725	0.2627792
1.7625	0.28598333
1.8	0.30033324
1.8375	0.34048641
1.875	0.36163741
1.9125	0.37990438
1.95	0.3949334
1.9875	0.40999731
2.025	0.43786287
2.0625	0.45270585
2.1	0.46271555
2.1375	0.40499116
2.175	0.41188695
2.2125	0.31322599
2.25	0.24384137
2.2875	0.22609376
2.325	0.22832288
2.3625	0.1971288
2.4	0.19515633
2.4375	0.47423152
2.475	0.3559208
2.5125	0.07721943
