# Supplementary material for: TC10 regulates breast cancer invasion and metastasis by controlling membrane type-1 matrix metalloproteinase at invadopodia
Source: Commun Biol. 2021 Sep 16;4:1091. doi: 10.1038/s42003-021-02583-3 (PMC8445963; doi:10.1038/s42003-021-02583-3)
Supplement: Supplementary file 9 — Supplementary Data2 [file 42003_2021_2583_MOESM9_ESM.pdf]

Supplementary Data 2:

TC10 FRET biosensor sequence:

mCerulean-cpmVenus version:

mCerulean1 (Syn.Mod.):
ATGGTGTCCAAAGGAGAAGAACTGTTTACAGGAGTGGTCCCTATTCTGGTGGAACTGGAT
GGAGATGTGAATGGACATAAATTTTCCGTGAGCGGAGAAGGAGAAGGAGACGCTACATAT
GGAAAACTGACACTGAAGTTTATTTGTACAACAGGAAAACTGCCTGTGCCTTGGCCTACA
CTGGTGACCACACTGACATGGGGAGTCCAGTGTTTTGCTAGGTATCCTGATCATATGAAA
CAGCATGATTTCTTTAAAAGCGCTATGCCTGAGGGATATGTGCAGGAAAGGACAATTTTC
TTTAAAGATGATGGAAATTATAAAACAAGGGCTGAAGTGAAATTTGAAGGAGATACACTG
GTGAATAGGATTGAACTGAAAGGAATTGATTTTAAAGAAGATGGAAATATTCTGGGACAT
AAACTGGAATATAATGCTATTAGCGACAATGTGTACATTACAGCTGATAAACAGAAAAAT
GGAATTAAGGCTAATTTTAAAATTAGGCATAATATTGAAGATGGAAGCGTGCAGCTGGCT
GATCATTATCAGCAGAATACACCTATTGGAGATGGACCTGTGCTGCTGCCTGATAATCAT
TATCTGTCCACACAGAGCAAACTGTCCAAAGATCCTAATGAAAAAAGGGACCATATGGTG
CTGCTGGAATTTGTGACAGCTGCCGGCATTACCCTGGGAATGGATGAACTGTATAAA

Linker:
GGATCC

PBD1:
AAGGAGCGCCCCGAGATTTCCCTCCCTTCCGATTTCGAACACACGATTCACGTCGGGTTC
GATGCTGTCACGGGGGAGTTCACGGGGATGCCTGAGCAGTGGGCCCGCCTGCTCCAGACG
TCCAACATCACGAAGTCCGAGCAGAAGAAAAACCCTCAGGCTGTCCTGGATGTCCTCGAA
TTTTACAACTCCAAGAAAACGTCCAACAGCCAGAAATACATGAGCTTCACGGATAAGTCC

Linker:
GGCAGCGGCGGCAAGCTTCCCCCCGGCAGCGGGGGCTCCGGC

PBD2 with H83/86D:
AAGGAACGGCCTGAAATCAGCCTGCCCAGCGACTTCGAGGACACCATCGATGTGGGCTTC
GACGCCGTGACCGGCGAGTTTACCGGCATGCCCGAACAGTGGGCTCGGCTCCTGCAGACC
AGCAACATCACCAAAAGCGAACAGAAAAAGAACCCCCAGGCCGTGCTGGACGTGCTGGAG
TTCTATAATAGCAAAAAGACCAGCAATTCCCAGAAGTATATGTCCTTCACCGACAAAAGC

Linker:
GCGGCCGCA

mcp229Venus:
ATGGGCAGCACCAGCGGCAGCGGCAAACCGGGCAGCGGCGAAGGCAGCATGGTGAGCAAG
GGCGAGGAGCTGTTCACCGGGGTGGTGCCCATCCTGGTCGAGCTGGACGGCGACGTAAAC
GGCCACAAGTTCAGCGTGTCCGGCGAGGGCGAGGGCGATGCCACCTACGGCAAGCTGACC
CTGAAGCTGATCTGCACCACCGGCAAGCTGCCCGTGCCCTGGCCCACCCTCGTGACCACC
CTGGGCTACGGCCTGATGTGCTTCGCCCGCTACCCCGACCACATGAAGCAGCACGACTTC

TTCAAGTCCGCCATGCCCGAAGGCTACGTCCAGGAGCGCACCATCTTCTTCAAGGACGAC
GGCAACTACAAGACCCGCGCCGAGGTGAAGTTCGAGGGCGACACCCTGGTGAACCGCATC
GAGCTGAAGGGCATCGACTTCAAGGAGGACGGCAACATCCTGGGGCACAAGCTGGAGTAC
AACTACAACAGCCACAACGTCTATATCACCGCCGACAAGCAGAAGAACGGCATCAAGGCC
AACTTCAAGATCCGCCACAACATCGAGGACGGCGGCGTGCAGCTCGCCGACCACTACCAG
CAGAACACCCCCATCGGCGACGGCCCCGTGCTGCTGCCCGACAACCACTACCTGAGCTAC
CAGTCCAAGCTGAGCAAAGACCCCAACGAGAAGCGCGATCACATGGTCCTGCTGGAGTTC
GTGACCGCCGCCGGG

Linker:
GAATTC

TC10:
ATGCCCGGAGCCGGCCGCAGCAGCATGGCTCACGGGCCCGGCGCGCTGATGCTCAAGTGC
GTGGTGGTCGGCGACGGGGCGGTGGGCAAGACGTGCCTACTCATGAGCTATGCCAACGAC
GCCTTCCCGGAGGAGTACGTGCCCACCGTCTTCGACCACTACGCAGTCAGCGTCACCGTG
GGGGGCAAGCAGTACCTCCTAGGACTCTATGACACGGCCGGACAGGAAGACTATGACCGT
CTGAGGCCTTTATCTTACCCAATGACCGATGTCTTCCTTATATGCTTCTCGGTGGTAAAT
CCAGCCTCATTTCAAAATGTGAAAGAGGAGTGGGTACCGGAACTTAAGGAATACGCACCA
AATGTACCCTTTTTATTAATAGGAACTCAGATTGATCTCCGAGATGACCCCAAAACTTTA
GCAAGACTGAATGATATGAAAGAAAAACCTATATGTGTGGAACAAGGACAGAAACTAGCA
AAAGAGATAGGAGCATGCTGCTATGTGGAATGTTCAGCTTTAACCCAGAAGGGATTGAAG
ACTGTTTTTGATGAGGCTATCATAGCCATTTTAACTCCAAAGAAACACACTGTAAAAAAA
AGAATAGGATCAAGATGTATAAACTGTTGTTTAATTACGTGA

Near infrared version:

miRFP720:
ATGGCCGAGGGCAGCGTGGCCCGCCAGCCCGACCTGCTGACCTGCGACGACGAGCCCATC
CACATCCCCGGCGCCATCCAGCCCCACGGCCTGCTGCTGGCCCTGGCCGCCGACATGACC
ATCGTGGCCGGCAGCGACAACCTGCCCGAGCTGACCGGCCTGGCCATCGGCGCCCTGATC
GGCCGCAGCGCCGCCGACGTGTTCGACAGCGAGACCCACAACCGCCTGACCATCGCCCTG
GCCGAGCCCGGCGCCGCCGTGGGCGCCCCCATCACCGTGGGCTTCACCATGCGCAAGGAC
GCCGGCTTCATCGGCAGCTGGCACCGCCACGACCAGCTGATCTTCCTGGAGCTGGAGCCC
CCCCAGCGCGACGTGGCCGAGCCCCAGGCCTTCTTCCGCCGCACCAACAGCGCCATCCGC
CGCCTGCAGGCCGCCGAGACCCTGGAGAGCGCCTGCGCCGCCGCCGCCCAGGAGGTGCGC
AAGATCACCGGCTTCGACCGCGTGATGATCTACCGCTTCGCCAGCGACTTCAGCGGCAGC
GTGATCGCCGAGGACCGCTGCGCCGAGGTGGAGAGCAAGCTGGGCCTGCACTACCCCGCC
AGCTTCATCCCCGCCCAGGCCCGCCGCCTGTACACCATCAACCCCGTGCGCATCATCCCC
GACATCAACTACCGCCCCGTGCCCGTGACCCCCGACCTGAACCCCGTGACCGGCCGCCCC
ATCGACCTGAGCTTCGCCATCCTGCGCAGCGTGAGCCCCAACCACCTGGAGTTCATGCGC
AACATCGGCATGCACGGCACCATGAGCATCAGCATCCTGCGCGGCGAGCGCCTGTGGGGC
CTGATCGTGTGCCACCACCGCACCCCCTACTACGTGGACCTGGACGGCCGCCAGGCCTGC
AAGCGCGTGGCCGAGCGCCTGGCCACCCAGATCGGCGTGATGGAGGAG

Linker:

GGATCC

PBD1:
AAGGAGCGCCCCGAGATTTCCCTCCCTTCCGATTTCGAACACACGATTCACGTCGGGTTC
GATGCTGTCACGGGGGAGTTCACGGGGATGCCTGAGCAGTGGGCCCGCCTGCTCCAGACG
TCCAACATCACGAAGTCCGAGCAGAAGAAAAACCCTCAGGCTGTCCTGGATGTCCTCGAA
TTTTACAACTCCAAGAAAACGTCCAACAGCCAGAAATACATGAGCTTCACGGATAAGTCC

Linker:
GGCAGCGGCGGCAAGCTTCCCCCCGGCAGCGGGGGCTCCGGC

PBD2 with H83/86D:
AAGGAACGGCCTGAAATCAGCCTGCCCAGCGACTTCGAGGACACCATCGATGTGGGCTTC
GACGCCGTGACCGGCGAGTTTACCGGCATGCCCGAACAGTGGGCTCGGCTCCTGCAGACC
AGCAACATCACCAAAAGCGAACAGAAAAAGAACCCCCAGGCCGTGCTGGACGTGCTGGAG
TTCTATAATAGCAAAAAGACCAGCAATTCCCAGAAGTATATGTCCTTCACCGACAAAAGC

Linker:
GCGGCCGGCACGTCTGGCTCCGGGAAAGGCAGCGGGGAAGGCTCCACCAAGGGGACCTCC
GGGAGCGGCAAGGGGTCCGGCGAGGGAAGCACGAAAGGCGGCAGCGCTGCCGGCACATCT
GGAAGCGGCAAGGGCTCTGGGGAGGGGTCCACTAAAGGAGGGAGCGCGGCCGCT

miRFP670:
ATGGTGGCCGGCCACGCCAGCGGCAGCCCCGCCTTCGGCACCGCCAGCCACAGCAACTGC
GAGCACGAGGAGATCCACCTGGCCGGCAGCATCCAGCCCCACGGCGCCCTGCTGGTGGTG
AGCGAGCACGACCACCGCGTGATCCAGGCCAGCGCCAACGCCGCCGAGTTCCTGAACCTG
GGCAGCGTGCTGGGCGTGCCCCTGGCCGAGATCGACGGCGACCTGCTGATCAAGATCCTG
CCCCACCTGGACCCCACCGCCGAGGGCATGCCCGTGGCCGTGCGCTGCCGCATCGGCAAC
CCCAGCACCGAGTACTGCGGCCTGATGCACCGCCCCCCGAGGGCGGCCTGATCATCGAG
CTGGAGCGCGCCGGCCCCAGCATCGACCTGAGCGGCACCCTGGCCCCCGCCCTGGAGCGC
ATCCGCACCGCCGGCAGCCTGCGCGCCCTGTGCGACGACACCGTGCTGCTGTTCCAGCAG
TGCACCGGCTACGACCGCGTGATGGTGTACCGCTTCGACGAGCAGGGCCACGGCCTGGTG
TTCAGCGAGTGCCACGTGCCCGGCCTGGAGAGCTACTTCGGCAACCGCTACCCCAGCAGC
ACCGTGCCCCAGATGGCCCGCCAGCTGTACGTGCGCCAGCGCGTGCGCGTGCTGGTGGAC
GTGACCTACCAGCCCGTGCCCCTGGAGCCCCGCCTGAGCCCCCTGACCGGCCGCGACCTG
GACATGAGCGGCTGCTTCCTGCGCAGCATGAGCCCCTGCCACCTGCAGTTCCTGAAGGAC
ATGGGCGTGCGCGCCACCCTGGCCGTGAGCCTGGTGGTGGGCGGCAAGCTGTGGGGCCTG
GTGGTGTGCCACCACTACCTGCCCCGCTTCATCCGCTTCGAGCTGCGCGCCATCTGCAAG
CGCCTGGCCGAGCGCATCGCCACCCGCATCACCGCCCTGGAGAGC

Linker:
GGCAGCGGCTCCGGGAGCGGGTCCGGAGGCGAATTC:

TC10:
ATGCCCGGAGCCGGCCGCAGCAGCATGGCTCACGGGCCCGGCGCGCTGATGCTCAAGTGC
GTGGTGGTCGGCGACGGGGCGGTGGGCAAGACGTGCCTACTCATGAGCTATGCCAACGAC
GCCTTCCCGGAGGAGTACGTGCCCACCGTCTTCGACCACTACGCAGTCAGCGTCACCGTG

GGGGGCAAGCAGTACCTCCTAGGACTCTATGACACGGCCGGACAGGAAGACTATGACCGT
CTGAGGCCTTTATCTTACCCAATGACCGATGTCTTCCTTATATGCTTCTCGGTGGTAAAT
CCAGCCTCATTTCAAAATGTGAAAGAGGAGTGGGTACCGGAACTTAAGGAATACGCACCA
AATGTACCCTTTTTATTAATAGGAACTCAGATTGATCTCCGAGATGACCCCAAAACTTTA
GCAAGACTGAATGATATGAAAGAAAAACCTATATGTGTGGAACAAGGACAGAAACTAGCA
AAAGAGATAGGAGCATGCTGCTATGTGGAATGTTCAGCTTTAACCCAGAAGGGATTGAAG
ACTGTTTTTGATGAGGCTATCATAGCCATTTTAACTCCAAAGAAACACACTGTAAAAAAA
AGAATAGGATCAAGATGTATAAACTGTTGTTTAATTACGTGA
