# Supplementary material for: TC10 regulates breast cancer invasion and metastasis by controlling membrane type-1 matrix metalloproteinase at invadopodia
Source: Commun Biol. 2021 Sep 16;4:1091. doi: 10.1038/s42003-021-02583-3 (PMC8445963; doi:10.1038/s42003-021-02583-3)
Supplement: Supplementary file 11 — Reporting Summary [file 42003_2021_2583_MOESM11_ESM.pdf]

nature portfolio

Corresponding author(s): Louis Hodgson
Last updated by author(s): Jul 29, 2021

# Reporting Summary

Nature Portfolio wishes to improve the reproducibility of the work that we publish. This form provides structure for consistency and transparency in reporting. For further information on Nature Portfolio policies, see our Editorial Policies and the Editorial Policy Checklist.

## Statistics

For all statistical analyses, confirm that the following items are present in the figure legend, table legend, main text, or Methods section.

| n/a | Confirmed | |
|---|---|---|
| ☐ | ☒ | The exact sample size ($n$) for each experimental group/condition, given as a discrete number and unit of measurement |
| ☐ | ☒ | A statement on whether measurements were taken from distinct samples or whether the same sample was measured repeatedly |
| ☐ | ☒ | The statistical test(s) used AND whether they are one- or two-sided<br>*Only common tests should be described solely by name; describe more complex techniques in the Methods section.* |
| ☒ | ☐ | A description of all covariates tested |
| ☐ | ☒ | A description of any assumptions or corrections, such as tests of normality and adjustment for multiple comparisons |
| ☐ | ☒ | A full description of the statistical parameters including central tendency (e.g. means) or other basic estimates (e.g. regression coefficient) AND variation (e.g. standard deviation) or associated estimates of uncertainty (e.g. confidence intervals) |
| ☐ | ☒ | For null hypothesis testing, the test statistic (e.g. $F$, $t$, $r$) with confidence intervals, effect sizes, degrees of freedom and $P$ value noted<br>*Give P values as exact values whenever suitable.* |
| ☒ | ☐ | For Bayesian analysis, information on the choice of priors and Markov chain Monte Carlo settings |
| ☒ | ☐ | For hierarchical and complex designs, identification of the appropriate level for tests and full reporting of outcomes |
| ☒ | ☐ | Estimates of effect sizes (e.g. Cohen's $d$, Pearson's $r$), indicating how they were calculated |

*Our web collection on statistics for biologists contains articles on many of the points above.*

## Software and code

Policy information about availability of computer code

| | |
|---|---|
| Data collection | Metamorph version 7.10.3.297 software; Visview version 5.0.0.8 software |
| Data analysis | Matlab version 2011a software; All Matlab codes and Metamorph scripts used were previously published elsewhere, but are also available from the corresponding author on request. |

For manuscripts utilizing custom algorithms or software that are central to the research but not yet described in published literature, software must be made available to editors and reviewers. We strongly encourage code deposition in a community repository (e.g. GitHub). See the Nature Portfolio guidelines for submitting code & software for further information.

## Data

Policy information about availability of data

All manuscripts must include a data availability statement. This statement should provide the following information, where applicable:

- Accession codes, unique identifiers, or web links for publicly available datasets
- A description of any restrictions on data availability
- For clinical datasets or third party data, please ensure that the statement adheres to our policy

The data that support the findings of this study are available from the corresponding author on request.

# Field-specific reporting

Please select the one below that is the best fit for your research. If you are not sure, read the appropriate sections before making your selection.

[x] Life sciences [ ] Behavioural & social sciences [ ] Ecological, evolutionary & environmental sciences

For a reference copy of the document with all sections, see nature.com/documents/nr-reporting-summary-flat.pdf

# Life sciences study design

All studies must disclose on these points even when the disclosure is negative.

| | |
|---|---|
| Sample size | No sample size determination calculations were done. All experiments were performed for at least n=3 or greater independent experiments. |
| Data exclusions | Data from an entire experiment set were excluded only when the matched control experiments did not show reproducibility of the previously observed and established/published results. |
| Replication | Data replication were confirmed by comparing the matched control results to the previously published/established results and confirmed reproducibility across studies. |
| Randomization | No data randomizations were involved or performed |
| Blinding | Investigators were not blinded. The blinding was not performed as these were traditional cell biology and molecular biology assays. |

# Reporting for specific materials, systems and methods

We require information from authors about some types of materials, experimental systems and methods used in many studies. Here, indicate whether each material, system or method listed is relevant to your study. If you are not sure if a list item applies to your research, read the appropriate section before selecting a response.

## Materials & experimental systems

| n/a | Involved in the study | |
|---|---|---|
| [ ] | [x] | Antibodies |
| [ ] | [x] | Eukaryotic cell lines |
| [x] | [ ] | Palaeontology and archaeology |
| [ ] | [x] | Animals and other organisms |
| [x] | [ ] | Human research participants |
| [x] | [ ] | Clinical data |
| [x] | [ ] | Dual use research of concern |

## Methods

| n/a | Involved in the study | |
|---|---|---|
| [x] | [ ] | ChIP-seq |
| [x] | [ ] | Flow cytometry |
| [x] | [ ] | MRI-based neuroimaging |

## Antibodies

| | |
|---|---|
| Antibodies used | TC10 (Novus, Littleton, CO, USA; 07-2151; rabbit polyclonal used at 1:500 for western blots), p190 (BD Transduction Laboratories; 610149; Clone 30/p190; mouse monoclonal), p120 (Abcam, Cambridge, UK; ab2922; Clone B4F8; mouse monoclonal), Exo70 (Santa Cruz Biotechnology; sc-365825; Clone D-6; mouse monoclonal), Vamp7 (Abcam; ab36195; Clone 158.2; mouse monoclonal), MT1-MMP-Hinge region (Millipore, Burlington, MA, USA; AB6004; rabbit polyclonal), MT1-MMP (Millipore; MAB3328; Clone LEM-2/15.8; mouse monoclonal), MT1-MMP (Abcam; ab38971; rabbit polyclonal), Cortactin (Abcam; ab3333; Clone 0.T.21; mouse monoclonal, used at 1:600), Cortactin (Abcam; ab81208; Clone EP1922Y; rabbit monoclonal), Cortactin (Santa Cruz Biotechnology; sc-30771; G-18; goat polyclonal), MYC (Cell Signaling Technology, Danvers, MA, USA; mab2278; Clone 71D10; rabbit monoclonal), FLAG (Sigma; F1804; Clone M2; mouse monoclonal), EGFP (Roche; 11814460001; Clones 7.1 and 13.1; mixture mouse monoclonal), Rac1 (Millipore; 05-389; Clone 23A8; mouse monoclonal), Cdc42 (Santa Cruz Biotechnology; sc-8401; Clone B-8; mouse monoclonal), and RhoA (Santa Cruz Biotechnology; sc-418; Clone 26C4; mouse monoclonal). Unless otherwise stated, all primary antibodies were used at 1:200 dilution for immunofluorescence and 1:1,000 for western blotting. |
| Validation | Validation statements for all of the commercial antibodies were as provided by the manufacturers. We also provide validation of the TC10 antibody against purified TC10 vs Cdc42 in the supplementary figure. |

## Eukaryotic cell lines

Policy information about cell lines

| | |
|---|---|
| Cell line source(s) | MTLn3 (Jeffrey Segall, PhD); MDA-MB-231 (ATCC); HEK293T(ATCC) |
| Authentication | Cell lines were not independently authenticated |

| | |
|---|---|
| Mycoplasma contamination | Cells were tested for mycoplasma contamination routinely via PCR-based methods (Stratagene kit). |
| Commonly misidentified lines (See ICLAC register) | N/A |

## Animals and other organisms

Policy information about studies involving animals; ARRIVE guidelines recommended for reporting animal research

| | |
|---|---|
| Laboratory animals | Mouse; SCID, female; 6-8 week old |
| Wild animals | N/A |
| Field-collected samples | N/A |
| Ethics oversight | Institutional Animal Care and Use Committee of the Albert Einstein College of Medicine (protocol 20170507) |

Note that full information on the approval of the study protocol must also be provided in the manuscript.
